# Microtubules control cellular shape and coherence in amoeboid migrating cells

**DOI:** 10.1083/jcb.201907154

**Published:** 2020-05-07

**Authors:** Aglaja Kopf, Jörg Renkawitz, Robert Hauschild, Irute Girkontaite, Kerry Tedford, Jack Merrin, Oliver Thorn-Seshold, Dirk Trauner, Hans Häcker, Klaus-Dieter Fischer, Eva Kiermaier, Michael Sixt

**Affiliations:** 1 Institute of Science and Technology Austria, Klosterneuburg, Austria; 2 Biomedical Center, Walter Brendel Center of Experimental Medicine, Institute of Cardiovascular Physiology and Pathophysiology, University Hospital, Ludwig-Maximilians University of Munich, Munich, Germany; 3 Department of Immunology, State Research Institute Centre for Innovative Medicine, Vilnius, Lithuania; 4 Institute of Biochemistry and Cell Biology, Medical Faculty, Otto-von-Guericke University, Magdeburg, Germany; 5 Department of Pharmacy, Ludwig-Maximilians University of Munich, Munich, Germany; 6 Department of Chemistry, New York University, New York, NY; 7 Division of Microbiology and Immunology, Department of Pathology, University of Utah, Salt Lake City, UT; 8 Life and Medical Sciences Institute (LIMES), Immune and Tumor Biology, University of Bonn, Bonn, Germany

## Abstract

Cells navigating through complex tissues face a fundamental challenge: while multiple protrusions explore different paths, the cell needs to avoid entanglement. How a cell surveys and then corrects its own shape is poorly understood. Here, we demonstrate that spatially distinct microtubule dynamics regulate amoeboid cell migration by locally promoting the retraction of protrusions. In migrating dendritic cells, local microtubule depolymerization within protrusions remote from the microtubule organizing center triggers actomyosin contractility controlled by RhoA and its exchange factor Lfc. Depletion of Lfc leads to aberrant myosin localization, thereby causing two effects that rate-limit locomotion: (1) impaired cell edge coordination during path finding and (2) defective adhesion resolution. Compromised shape control is particularly hindering in geometrically complex microenvironments, where it leads to entanglement and ultimately fragmentation of the cell body. We thus demonstrate that microtubules can act as a proprioceptive device: they sense cell shape and control actomyosin retraction to sustain cellular coherence.

## Teaser

When cells migrate in complex environments, the dynamics of ramified protrusions need to be tightly coordinated to prevent rupture of the cell body. Kopf et al. show in dendritic cells that microtubules, via the RhoA exchange factor Lfc, prevent fragmentation by locally regulating actomyosin contractility.

## Introduction

How different cell types maintain their typical shape and how cells with a dynamic shape prevent loss of physical coherence are poorly understood. This issue becomes particularly critical in migrating cells, in which protrusion of the leading edge has to be balanced by retraction of the tail (Xu et al., 2003; Tsai et al., 2019) and where multiple protrusions of one cell often compete for dominance, as exemplified in the split pseudopod model of chemotactic migration (Insall, 2010; Andrew and Insall, 2007). The two prevalent models of how remote edges of mammalian cells communicate with each other are based on the sensing of endogenous mechanical parameters that, in turn, control the actomyosin system. In cell types that tightly adhere to substrates via focal adhesion complexes, it has been proposed that actomyosin itself is the sensing structure and that adhesion sites communicate mechanically via actin stress fibers. When contractile stress fibers were pharmacologically, physically, or genetically perturbed in mesenchymal cells, the cells lost their coherent shape and spread in an uncontrolled manner (Cai et al., 2010; Cai and Sheetz, 2009). While communication via stress fibers is useful for adherent cells, it is unlikely to control the shape of amoeboid cells, which are often loosely adherent or nonadherent and accordingly do not assemble stress fibers (Friedl and Wolf, 2010; Valerius et al., 1981). A second model suggests that lateral plasma membrane tension, which is thought to rapidly equilibrate across the cell surface, mediates communication between competing protrusions and serves as an input system to control actomyosin dynamics (Diz-Muñoz et al., 2016; Houk et al., 2012; Keren et al., 2008; Murrell et al., 2015). While this principle has been convincingly demonstrated in small leukocytes such as neutrophil granulocytes moving in unconstrained environments, many amoeboid cells such as dendritic cells (DCs) are large and can adopt very ramified shapes (Friedl and Weigelin, 2008). Particularly when cells are tightly embedded in geometrically complex 3D matrices, it is questionable whether lateral membrane tension is able to equilibrate across the cell body (Shi et al., 2018). This raises the question of whether amoeboid cells maintain alternative systems that may act as a proprioceptive sense.

Any alternative “internal shape sensor” would need to operate across the cellular scale and mediate communication between cell edges often >100 µm apart. Centrally nucleated microtubules (MTs) seem well situated for such a function. We recently found that when leukocytes migrate through complex geometries, their nucleus acts as a mechanical gauge to lead them along the path of least resistance (Renkawitz et al., 2019). By spatial association with the nucleus, the microtubule organizing center (MTOC) and its nucleated MTs were involved in this navigational task, demonstrating that the positioning of the MTOC relative to the nucleus is critical for amoeboid navigation.

Here, we use DCs as an experimental paradigm to test the effects of the MT cytoskeleton on cell shape upon navigation in geometrically complex environments. DCs are the cellular link between innate and adaptive immunity. In their resting state, they seed peripheral tissues and sample the environment for immunogenic threats. Upon microbial encounter, they become activated, ingest pathogens, and differentiate into a mature state, which makes them responsive to chemokines binding to the chemokine receptor CCR7. CCR7 ligands guide DCs through the interstitium and via the afferent lymphatic vessels into the draining lymph node (Heuzé et al., 2013). Within lymph nodes, DCs present peripherally acquired antigens to naive T cells. DCs are an exquisite model for amoeboid navigation: they follow the global directional signal of a guidance cue while they locally adapt to the geometry of the interstitial matrix, without substantially remodeling or digesting their environment. We tested the mechanistic involvement of MTs in DC migration in reductionist and physiological environments.

## Results

### MTOC positioning and MT plus end dynamics determine the path of migration

**Figure S1.**
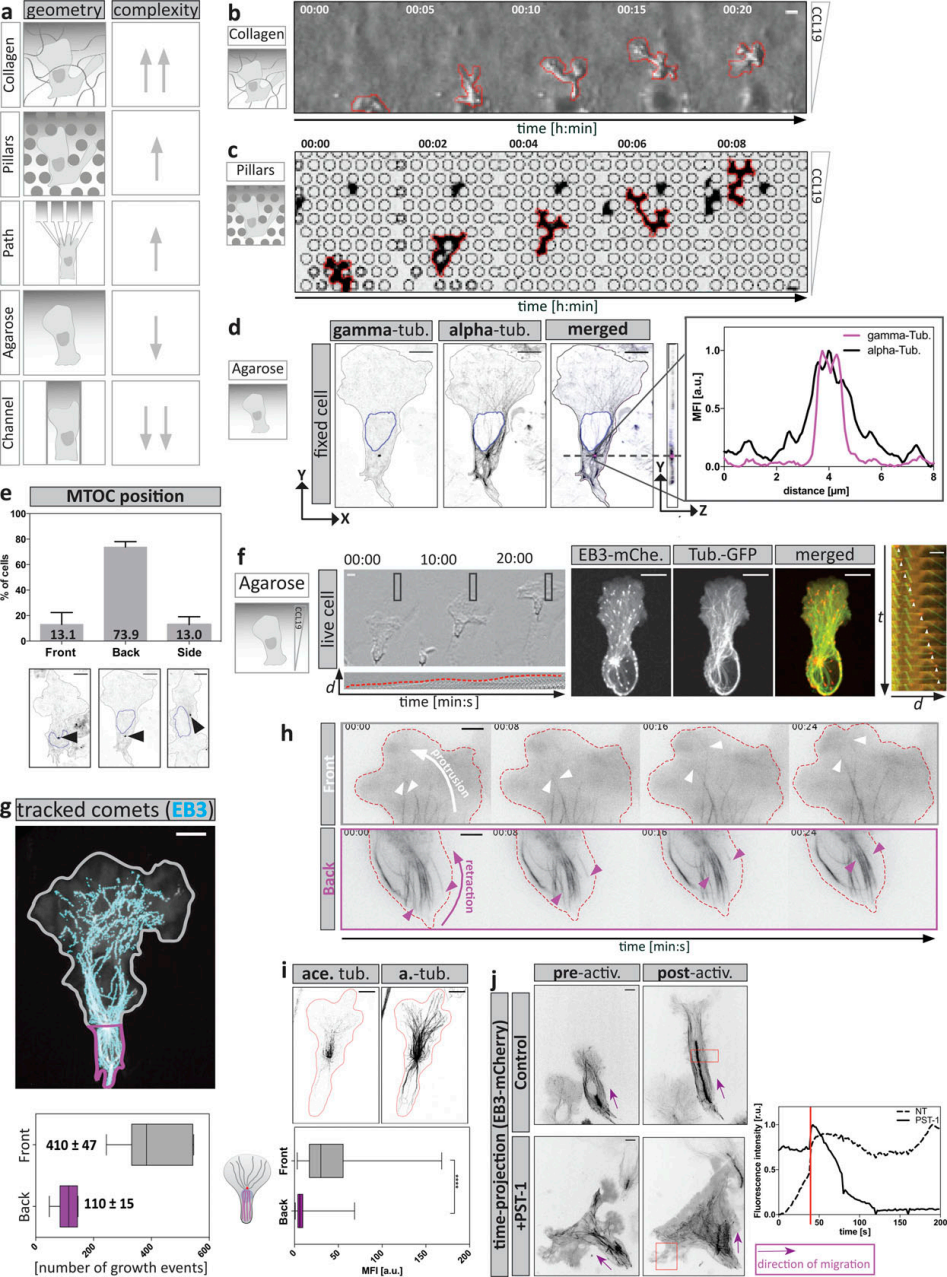
**DC migration within diverse matrices to study the role of the MT cytoskeleton during cell migration. (a)** Schematic representation of migration assays used in this study. Assays range from highly complex (top) and relatively uncontrollable geometries to very simple and precisely controllable PDMS-based structures (bottom). Complexity of the geometrical confinement correlates with dynamic shape changes of cells. Numbers of upward-facing arrows scale with high geometrical complexity and cell shape changes. Numbers of downward-facing arrows scale with low complexity. **(b)** Cell shape changes of a DC migrating in a collagen matrix along a soluble CCL19 gradient. Scale bar, 10 μm. **(c)** Dynamic cell shape changes are recapitulated during migration within a defined array of PDMS-based pillar structures. **(d)** MT nucleation from centrosomal origin determined by α- and γ-tubulin (tub.) staining. Right panel: The line profile of mean fluorescence intensities (MFI) along the purple line in the merged image is shown. Scale bar, 10 µm. **(e)** Determination of MTOC position by α- and γ-tubulin staining with respect to the nucleus. Black arrowheads indicate MTOC position. Mean ± SD of *n* = 256 cells from *N* = 3 experiments. **(f)** Double-reporter DC migrating under agarose along a soluble CCL19 gradient. Left panel: Cells migrating under agarose display a protrusive lamellipodium (lower panel: montage of boxed area) followed by a contractile trailing edge. Scale bar, 10 µm. Middle panel: EB3-mCherry (EB3-mChe.) localizes to the plus tips of tubulin-GFP–decorated MT filaments. Scale bar, 10 µm. Right panel: EB3-mCherry faithfully tracks growing MT filaments during DC migration. White arrowheads highlight the localization of EB3 signal at the tip of polymerizing tubulin filaments as the cell advances. Scale bar, 5 µm. **(g)** Automatically detected EB3 comets (cyan) overlaid on maximum intensity time projection (120 s) of an EB3-mcherry–expressing cell migrating under agarose. Lower panel: Quantification of MT growth events of front (gray) versus back (purple) directed MT tracks over a time period of 120 s of *n* = 7 cells. Boxes extend from 25th to 75th percentiles. Whiskers span minimum to maximum values. Scale bar, 10 µm. **(h)** Time-course analysis of MT filament dynamics of migrating DCs expressing EMTB-mCherry. Upper panel: Indicates the leading edge area. The white arrow represents membrane protrusion, and the white arrowheads represent elongating MT filaments. Lower panel: Indicates the trailing edge area in which the purple arrow represents membrane retraction and purple arrowheads MT filament depolymerization. Red dashed lines indicate cell edges. Scale bar, 10 µm. **(i)** Acetylated-tubulin (ace. tub.) staining in DCs fixed while migrating under agarose. Only cells with the MTOC localized in front of the nucleus were analyzed. Levels of acetylation were assessed by measuring the mean fluorescence intensity of acetylated-tubulin along individual α-tubulin (a.-tub) filaments (*n* = 87 filaments per condition from *N* = 3 experiments) directed toward the front (gray) or back (purple). Boxes extend from 25th to 75th percentiles. Whiskers span minimum to maximum values. Annotation above columns indicates results of unpaired Student’s *t* test; ****, P ≤ 0.0001. Scale bar, 10 µm. **(j)** EB3-mCherry localization of control or PST-1–treated cells migrating under agarose along a soluble CCL19 gradient. The red box indicates the photoactivated area magnified on the right. Magnified regions show time projection of EB3-mCherry intensities after local photoactivation. Purple arrows indicate the direction of cell migration. Lower panel on the right indicates fluorescence intensity evolution upon photoactivation of control or PST-1–treated cells. The red line highlights the time point of the initial photoactivation. Scale bar, 10 µm. activ., activation; NT, non-treated; r.u., relative units.

**Figure 1.**
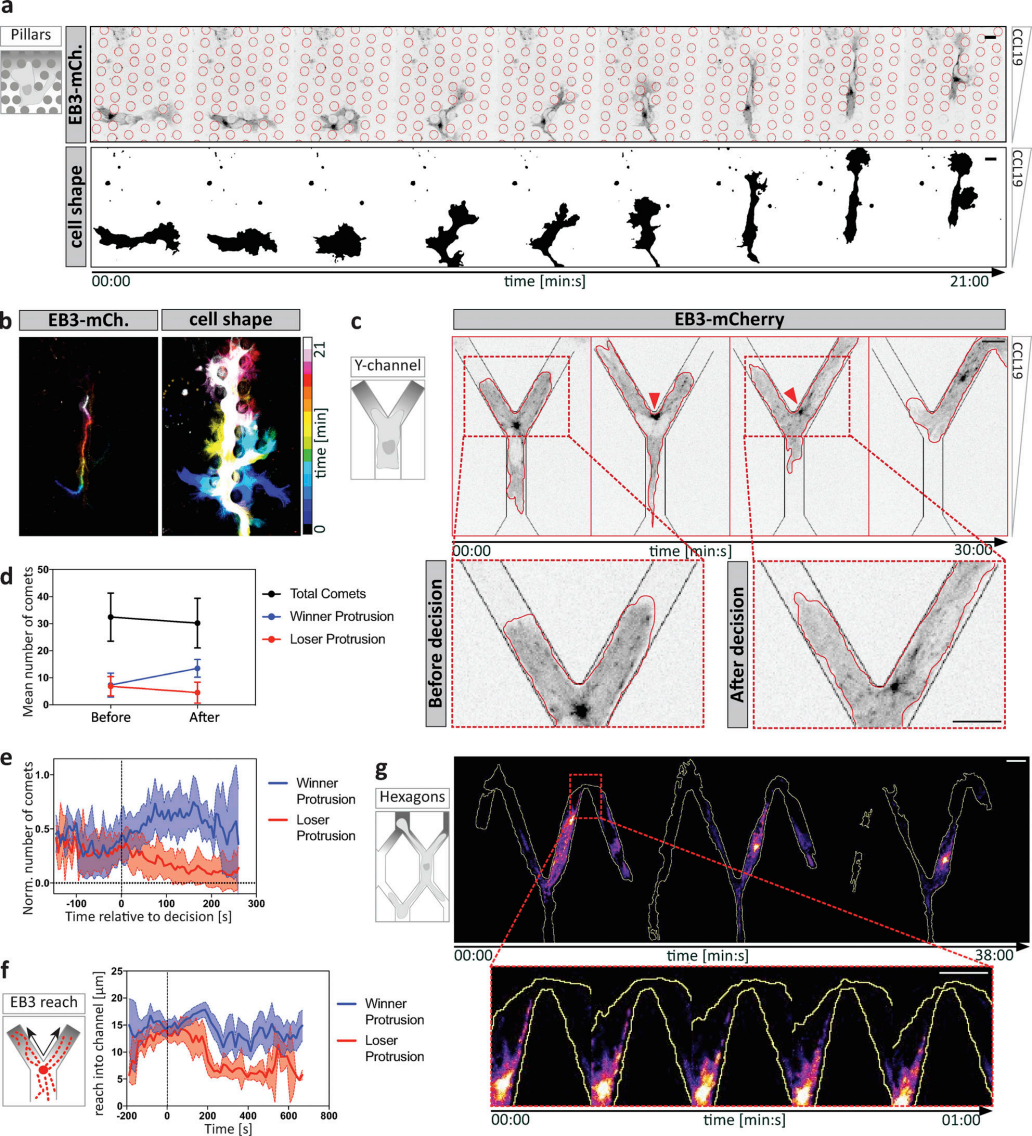
**MTOC positioning and MT dynamics determine the path of migration. (a)** DC migrating within a pillar array. Upper panel shows EB3-mCherry (EB3-mCh.) expression profile. Lower panel ooutlines dynamic cell shape changes. Scale bar, 10 µm. **(b)** Time projection of image sequence shown in panel a. Left panel indicates MTOC position over time. Right panel outlines the formation and retraction of multiple explorative protrusions over time. **(c)** DC migration in Y-shaped decision channels. Left panel outlines the channel geometry of Y-shaped devices. Upper panel shows the EB3-mCherry expression profile. Lower panel shows higher magnification at the decision point. Red arrowheads indicate MTOC position before and after the decision, respectively. Scale bar, 10 µm. **(d)** Mean number of EB3 comets in winner versus loser protrusions before and after the decision of *n* = 7 cells. Mean ± SD. **(e)** Mean number of EB3 comets normalized (Norm.) to the maximum number of comets in each protrusion over time of *n* = 7 cells ± SD. **(f)** Mean EB3 comet reach into protrusions over time of *n* = 7 cells ± SD. **(g)** EB3-mCherry–expressing DC migrating in a hexagonal channel array. Red box indicates area magnified in the lower panel. Scale bar, 10 µm. See also Video 1.

As cytoskeletal dynamics are notoriously difficult to visualize in situ or in physiological environments such as collagen gels, we used microfluidic pillar mazes (Renkawitz et al., 2018) as a reductionist setup that mimics some of the geometrical complexities of interstitial matrices while being accessible to imaging (Fig. S1, a–c). Within these devices, cells are confined between two closely adjacent surfaces, which are intersected by pillars of variable spacing. We exposed DCs differentiated from hematopoietic precursor cells to soluble gradients of the chemokine CCL19 (Redecke et al., 2013). To track MT plus ends, we generated precursor cell lines stably expressing end-binding protein 3 fused to mCherry (EB3-mCherry) and differentiated them into DCs. During time-lapse imaging, the MTOC was clearly detectable as the brightest spot radiating MT plus ends. This indicated that, in line with previous studies on different leukocyte subsets, MTs nucleate almost exclusively at the MTOC and that in a migrating DC, the MTOC is mainly located behind the nucleus (Fig. 1 a; and Fig. S1, d and e). When cells navigated through the pillar maze, the MTOC moved in a remarkably straight line up the chemokine gradient, although transient lateral protrusions regularly explored alternative paths between the pillars (Fig. 1 b). This observation was in line with the idea that the MTOC prescribes the path of the cell body and that lateral protrusions are retracted as soon as the MTOC passes through a gap.

To test how MT dynamics relate to MTOC positioning, we chemotactically guided DCs through Y-channels. In this configuration, migrating cells symmetrically extend protrusions into both channel arms before they stochastically retract one “loser protrusion” and, guided by the “winner protrusion,” pass through the other channel arm. Before the MTOC passed beyond the junction point, the number of EB3 comets was indistinguishable between both protrusions. Once the MTOC passed the junction point, the number of EB3 comets gradually decreased in the loser protrusion but increased in the winner protrusion (Fig. 1, c–e). We then quantified how far individual EB3 comets reached into the two protrusions and found that upon junction point passage of the MTOC, comets in the loser protrusion gradually decreased their distance traveled into the protrusion (Fig. 1 f). These findings led us to hypothesize that MTs, which grow in straight trajectories, eventually fail to enter curved or ramified protrusions. We therefore inserted migrating DCs into hexagonal arrays of interconnected channels and imaged EB3 dynamics. In this configuration, DCs exhibited multiple ramified and zigzag-shaped protrusions with 40° bending angles. These protrusions became less populated with growing plus tips as the MTOC advanced (Fig. 1 g and Video 1). This suggests that MTs are not able to sustain acute bending angles over long distances. Together, these observations suggest that whenever a protrusion turns into a retraction, this is accompanied by destabilization of MTs.

**Video 1.** **MT dynamics during migration within hexagonal channel arrays.** EB3-mCherry–expressing DC migrating within hexagonal channel array toward a soluble CCL19 gradient, acquired in 2-s intervals on an inverted spinning-disk microscope. Scale bar, 10 µm. Frame rate, 10 frames per second.

### Leading edge and trailing edge MTs show differential stability

**Figure 2.**
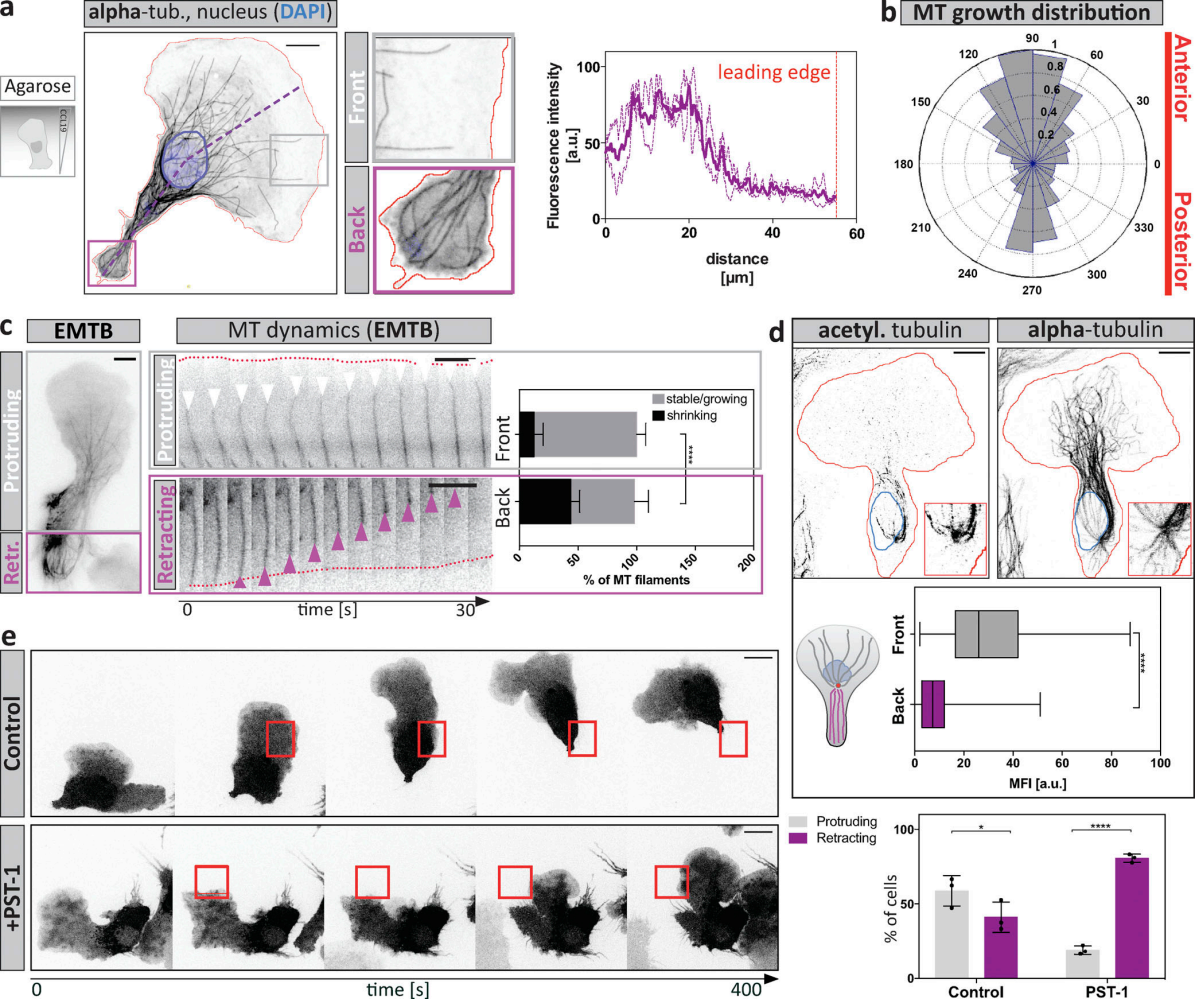
**MTs coordinate protrusion-retraction dynamics. (a)** Cells migrating under agarose along a soluble CCL19 gradient were fixed and stained for α-tubulin and the nucleus (DAPI). Boxed regions indicate trailing edge (purple) or pioneering (gray) MTs toward the leading edge. Right panel: Line profile of α-tubulin distribution along the anterior-posterior polarization axis, derived from the purple line in the left panel of *n* = 10 cells. Scale bar, 10 µm. **(b)** Angular distribution of automatically detected MT growth events according to EB3 signals along the anterior-posterior polarization axis. **(c)** MT dynamics during directed migration. EMTB-mCherry expressing DC migrating under a pad of agarose. Gray box indicates the protrusive cell front, whereas the purple boxed area denotes the contractile trailing edge. Growth (white arrowheads) and shrinkage (purple arrowheads) frequencies of individual MT filaments (according to EMTB labeling) were assessed in protrusive (front, white box) versus contractile (back, purple box) areas of the same migratory cell. Red dotted lines indicate cell edges. Growth events and catastrophes ≥1 µm were tracked for *n* = 10 filaments each in the respective region of *n* = 8 cells. Mean ± SD. Annotation above columns indicates results of unpaired Student’s *t* test; ****, P ≤ 0.0001. See also Video 2. Scale bar, 10 µm (left image) and 5 µm (right panels). **(d)** Acetylated (acetyl.)-tubulin staining in DCs fixed while migrating under agarose. Blue line indicates position of the nucleus; red line, cell outline. Insets show the area around the MTOC. Levels of acetylation were assessed by measuring the mean fluorescence intensity (MFI) of acetylated tubulin along individual α-tubulin filaments, directed toward the front (gray) or back (purple) of *n* = 87 filaments from *N* = 3 experiments. Boxes extend from 25th to 75th percentiles. Whiskers span minimum to maximum values. Annotation above columns indicates results of unpaired Student’s *t* test; ****, P ≤ 0.0001. Scale bar, 10 µm. **(e)** Time-lapse sequence of control or PST-1 treated cells stained with TAMRA, which were locally photoactivated (red boxes) during migration under agarose. Right panel indicates the frequency of local retractions upon photoactivation of control or PST-1–treated DCs during migration (*n* = 26 cells per condition ± SD from *N* = 3 experiments). Annotation above columns indicates results of unpaired Student’s *t* test; *, P ≤ 0.05, ****, P ≤ 0.0001. Scale bar, 10 µm. See also Video 3. Retr., retracting.

When confined in microfluidic devices, DCs are too thick to allow faithful long-term tracing of the entire MT population across the whole z volume. To capture individual MT dynamics, we therefore investigated DCs migrating along chemokine gradients when confined under a pad of agarose (Heit and Kubes, 2003). Here, the highly flattened morphology allows faithful tracing of fluorescent signals (Fig. S1 f). Under agarose, DCs migrate persistently and stably segregate into a protruding leading edge and a retracting trailing edge. We first mapped MTs in migrating DCs fixed under agarose. MTs polarized along the axis of migration, with highest signal intensities in trailing edge regions (Fig. 2 a) and few MTs protruding toward the leading edge (Fig. 2 a, gray inset). During migration, automated analysis of EB3 signal trajectories (Matov et al., 2010) showed that MT growth occurs across the entire cell area (Fig. S1, f and g), and the angular distribution revealed highly polarized growth along the anterior–posterior axis (Fig. 2 b). Turning to live cell migration, visualization of the MT binding domain of ensconsin (EMTB) revealed long-lived MTs at the leading lamellipodium, while MT dynamics were increased at the trailing edge, exhibiting higher frequencies of shrinkage events compared with front-directed filaments (Fig. 2 c, Fig. S1 h, and Video 2). To substantiate these findings, we stained fixed migratory DCs for the stabilizing acetylation modification and found that front-oriented, but not back-oriented, MTs were acetylated (Fig. 2 d), irrespective of MTOC positioning (Fig. S1 i). Together, these observations demonstrate that MT depolymerization is associated with cellular retraction in stably polarized as well as repolarizing cells.

**Video 2.** **MT dynamics in migratory DCs.** EMTB-mCherry–expressing DC migrating under agarose, acquired in 2-s intervals with TIRF microscopy (inverted signal). Left panel: Shows the protruding leading edge; black arrowheads indicate elongating MTs. Right panel: Shows retracting trailing edge of the same cell; purple arrowheads highlight shrinking MTs. Time in [min:s]. Scale bar, 5 µm. Frame rate, 5 frames per second.

### Local MT depolymerization causes local cellular retraction

We next tested for a possible causal relationship between MT depolymerization and retraction and devised a photo-pharmacological approach to depolymerize MTs in migratory cells with spatiotemporal control. We used photostatin-1 (PST-1), a reversibly photo-switchable analogue of combretastatin A-4, which can be functionally toggled between the active and inactive states by blue and green lights, respectively (Borowiak et al., 2015). To validate the approach, we locally activated the drug under simultaneous visualization of MT plus ends using EB3-mCherry. We found that local photoactivation triggered almost instantaneous disappearance of the EB3 signal in the presence but not in the absence of photostatin (Fig. S1 j), indicating rapid stalling of MT polymerization. Local depolymerization in protruding areas of the cell led to the consistent collapse of the illuminated protrusion and subsequent repolarization of the cell (Fig. 2 e and Video 3). This response was only observed in the presence of photostatin, while in the absence of the drug, cells were refractory to illumination. These data demonstrate a causal relationship between MT depolymerization and cellular retraction. This effect can act locally within a cell, raising the possibility that MTs coordinate subcellular retractions when navigating through geometrically complex environments such as collagen gels or a physiological interstitium.

**Video 3.** **Local MT depolymerization causes retraction.** TAMRA-stained DCs migrating under agarose were recorded every 2 s on an inverted spinning-disk microscope and locally photoactivated (red boxes) every 40 s using a 405-nm laser line. Cells were either untreated (left panel) or treated with the photo-switchable MT depolymerizing agent PST-1 (right panel). Time in [min:s]. Scale bar, 10 µm. Frame rate, 10 frames per second.

### MT depletion causes migratory failure due to hyperactive and destabilized actomyosin contractility

**Figure S2.**
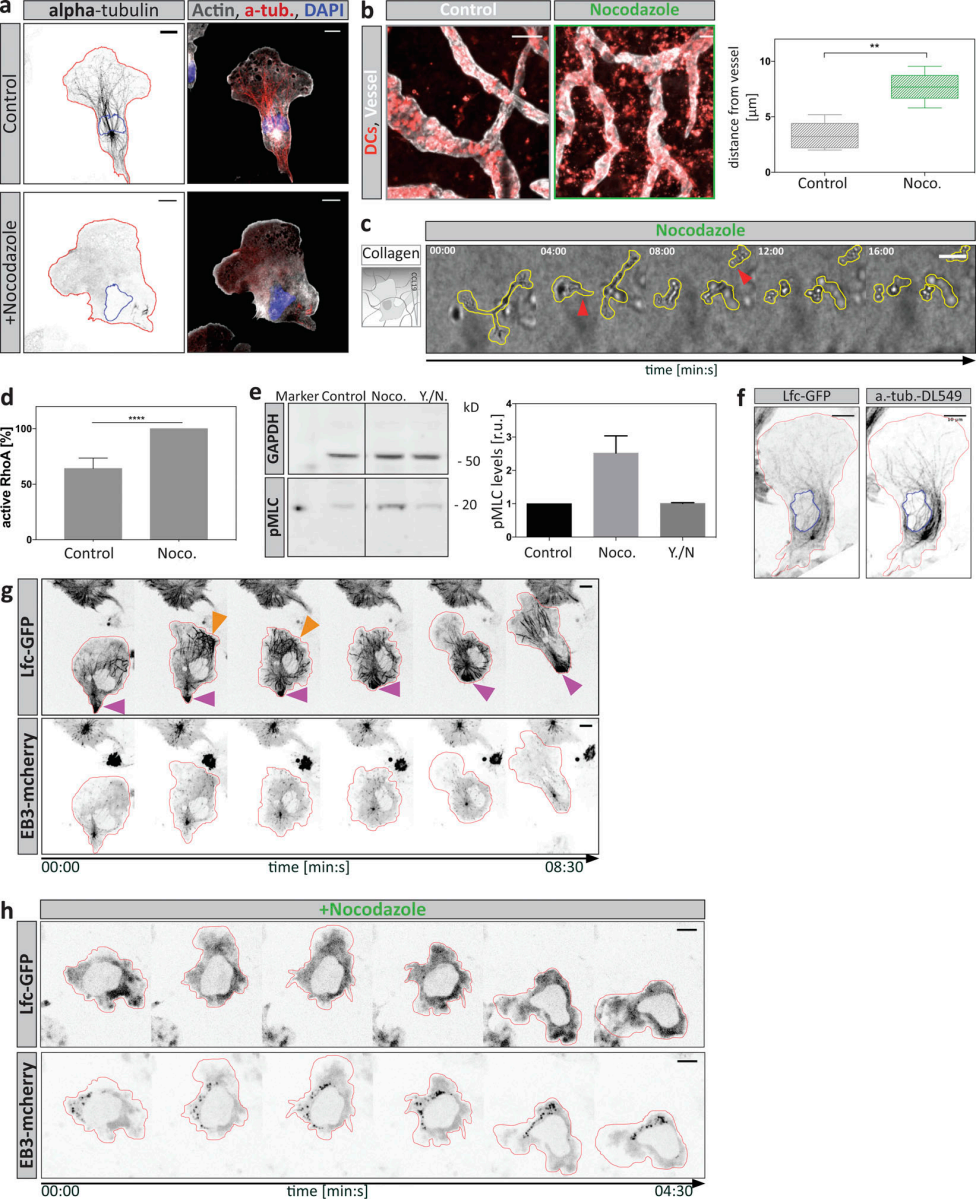
**Perturbation of the MT cytoskeleton affects DCs migration and subcellular Lfc localization. (a)** Non-treated control or nocodazole-treated cells migrating under agarose toward a CCL19 gradient were fixed and stained for endogenous distribution of α-tubulin and F-actin. Blue line indicates position of the nucleus; red line, cell outline. Scale bar, 10 µm. **(b)** In situ migration of endogenous DCs on a mouse ear sheet. Z-projections of separated ear sheets upon control conditions or nocodazole (Noco.) treatment. Lymphatic vessels were stained for Lyve-1 and DCs for MHC-II. Right panel: Mean distance from lymphatic vessels of endogenous DCs was determined 48 h after ear separation. Per condition, four mouse ears with two fields of view were analyzed. Boxes extend from 25th to 75th percentiles. Whiskers span minimum to maximum values. Annotation above columns indicates results of unpaired Student’s *t* test; **, P ≤ 0.01. Scale bar, 100 µm. **(c)** Nocodazole-treated DC migrating in a collagen gel toward a soluble CCL19 gradient. Yellow line outlines cell shape. Red arrowheads indicate the loss of cellular coherence. Scale bar, 100 µm. **(d)** Levels of active RhoA upon MT depolymerization with nocodazole determined by luminometry. RhoA activity levels were normalized to nocodazole-treated samples. Plotted is mean ± SD from *N* = 3 experiments. Annotation above columns indicates results of unpaired Student’s *t* test; ****, P ≤ 0.0001. **(e)** Levels of MLC phosphorylation determined by Western blot analysis. Cells were treated with the indicated compounds (DMSO, nocodazole [Noco.], or Y27632 together with nocodazole [Y./N.]). Right panel: The mean fluorescence intensity of phospho-MLC (pMLC) was normalized to the GAPDH signal and shown as fold increase relative to DMSO control ± SD. Blots are representative of *n* = 3 experiments. r.u., relative units. **(f)** Co-localization of Lfc-GFP on α-tubulin (a.-tub.) structures. An Lfc-GFP–expressing cell was fixed while migrating under agarose and stained for α-tubulin distribution. Scale bar, 10 µm. **(g)** Polarized distribution of Lfc-GFP in trailing edges and retracting protrusions. A double-reporter cell expressing Lfc-GFP and EB3-mcherry was followed while migrating under agarose. Purple arrowheads denote trailing edge, and orange arrowheads highlight retracting protrusion followed by cell repolarization. Scale bar, 10 µm. **(h)** Lfc-GFP distribution upon nocodazole treatment. A nocodazole-treated double–fluorescent reporter cell was followed while migrating under agarose. Note the absence of filamentous structures in both channels and the diffuse signal distribution of Lfc-GFP. Scale bar, 10 µm.

**Figure 3.**
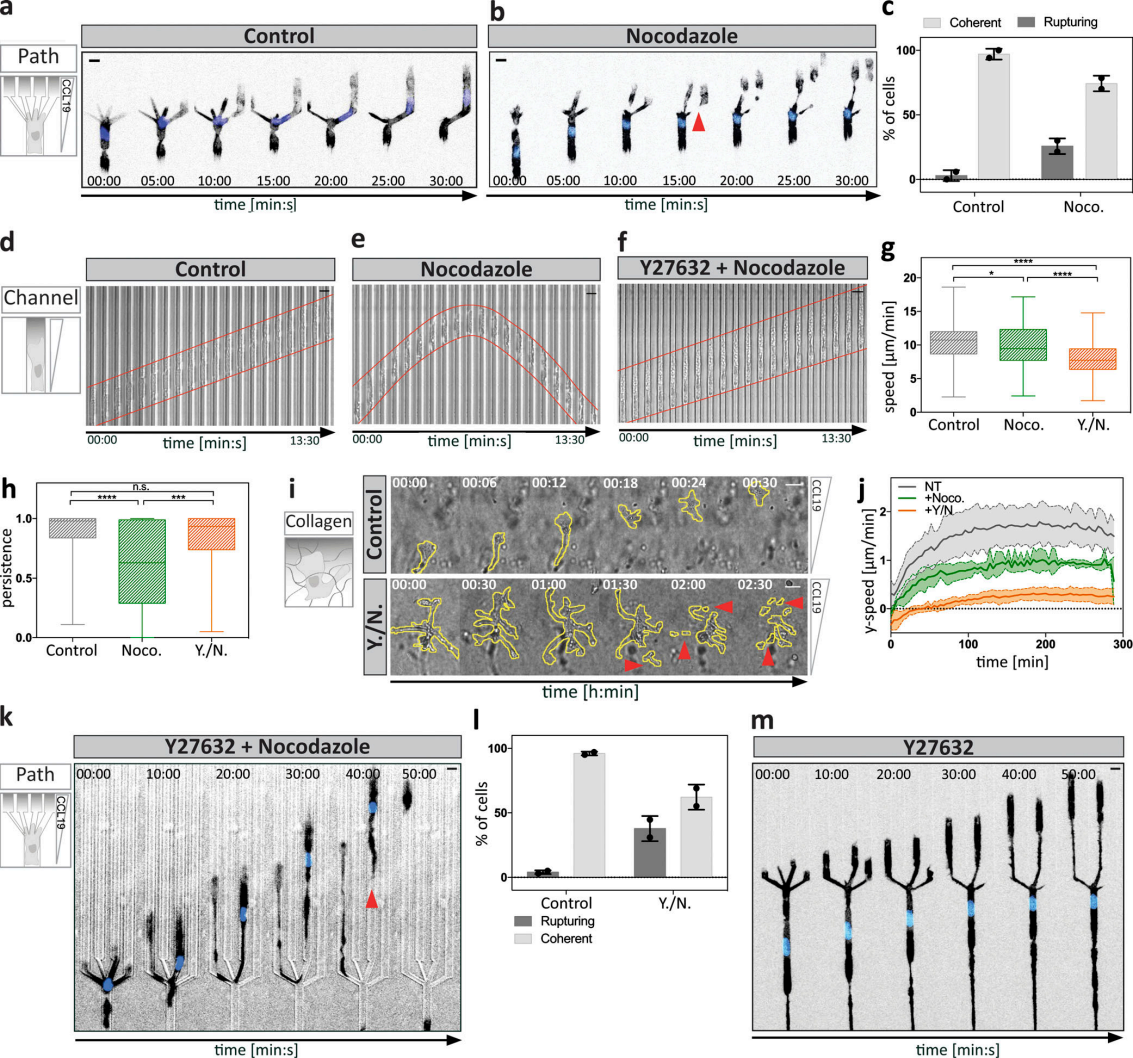
**MT depletion causes migratory failure due to hyperactive and destabilized actomyosin contractility. (a)** Lifeact-GFP–expressing DC migrating within a path choice device. Scale bar, 10 µm. **(b)** Nocodazole-treated Lifeact-GFP–expressing cell migrating within a path choice device. Note that the cell extends elongated protrusions into different channels. Red arrowhead denotes a cell rupturing event. See also Video 5. **(c)** Frequency of cell rupturing events during migration within path choice devices of *n* = 43 cells (control) and *n* = 44 cells (nocodazole; Noco.) ±SD of *N* = 2 experiments. **(d)** Time-lapse sequence of a cell migrating within a linear microchannel. See also Video 6. Scale bar, 10 μm. **(e)** Nocodazole-treated cell migrating in the same configuration as in d.****Scale bar, 10 μm. **(f)** Cell treated with a combination of Y27632 plus nocodazole migrating as shown in d. Scale bar, 10 µm. **(g)** Migration speed of control, nocodazole-treated, or double-treated cells using Y27632 and nocodazole within microchannels (*n* = minimum of 74 cells per condition from *N* = 4 experiments). Boxes extend from 25th to 75th percentiles. Whiskers span minimum to maximum values. Annotation above columns indicates results of one-way ANOVA with Tukey’s test; *, P ≤ 0.05; ****, P ≤ 0.0001. **(h)** Persistence of control, nocodazole-treated, or double-treated cells using Y27632 and nocodazole within microchannels (*n* = minimum of 74 cells per condition from *N* = 4 experiments). Boxes extend from 25th to 75th percentiles. Whiskers span minimum to maximum values. Annotation above columns indicates results of Kruskal-Wallis with Dunn’s test; ***, P ≤ 0.001; ****, P ≤ 0.0001. **(i)** DCs migrating within a collagen gel either non-treated (control) or double-treated with Y27632 and nocodazole. Note the different time intervals per condition. Red arrowheads indicate the loss of cellular coherence in the double-treated cell. Scale bar, 10 µm. See also Video 4. **(j)** Automated analysis of the y-directed speed of non-treated (NT), nocodazole-treated, or double-treated cells using Y27632 and nocodazole. Plot shows mean population migration velocities over time ± SD from *N* = 4 experiments. **(k)** Lifeact-GFP–expressing DC double-treated with Y27632 plus nocodazole migrating as in panel a. Red arrowhead denotes cell rupturing and loss of cellular coherence. Scale bar, 10 µm. **(l)** Frequency of cell rupturing events during migration within the path choice device of *n* = 40 cells (control) and *n* = 80 cells (Y./N.) ±SD of *N* = 2 experiments. **(m)** Lifeact-GFP–expressing DC treated with Y27632 migrating as in panel a. Note the extended protrusions are reaching far into separate channels without generating a productive decision within the indicated time. Scale bar, 10 µm. Noco., nocodazole; n.s., not significant; Y./N., double-treated with Y27632 and nocodazole.

Having established that local MT depolymerization causes cellular retraction, we next tested how the absence of MTs affects DC locomotion using nocodazole as an MT depolymerizing agent (Fig. S2 a). To test the contribution of MTs on DCs in their physiological environment, we first measured migration within explanted mouse ear skin preparations. Here, DCs failed to reach lymphatic vessels upon nocodazole treatment, while untreated cells efficiently approached and entered the vessels (Fig. S2 b). Similarly, when migrating in 3D collagen gels along gradients of chemokine, nocodazole-treated DCs were substantially impaired in their net movement toward the chemokine source. Notably, within collagen gels, DCs frequently lost coherence and fragmented upon nocodazole treatment (Fig. S2 c and Video 4). These observations pointed to defective coordination of retraction events. To more directly address this possibility, we used a microfluidic setup in which DCs migrated in a straight channel toward a junction where the channel split into four paths. In this setup, DCs initially inserted protrusions into all four channels, then retracted all but one protrusion and thereby selected the one path along which they advanced (Fig. 3 a and Fig. S1 a). The depletion of MTs with nocodazole led to uncoordinated protrusion dynamics and resulted in cell entanglement due to defective retraction of lateral protrusions (Fig. 3 b). Frequently, cells lost coherence when competing protrusions continued to migrate up the chemokine gradient until the cell ruptured into motile pieces (Fig. 3 c and Video 5). In contrast to these complex environments, in linear microfluidic channels MT depolymerization did not affect cell coherence (Fig. 3, d and e). In these geometrically simple environments in which uniaxial polarity is externally enforced and where there is no competition of multiple protrusions, nocodazole caused only a very minor reduction in locomotion speed (Fig. 3 g). While actual locomotion was intact upon nocodazole treatment, cells frequently changed direction, whereas untreated cells persistently moved through the channels (Fig. 3 h).

**Video 4.** **Perturbation of MT and myosin dynamics impairs DC migration in complex environments.** Mature DCs migrating along a soluble CCL19 gradient within a 3D collagen matrix. The montage shows separately acquired bright-field movies of control (DMSO), nocodazole-treated cells, and cells double-treated with Y27632 and nocodazole. Images were acquired every 60 s for 5 h and are represented as a single movie in 4-min intervals. Time in [min:s]. Scale bar, 100 µm for the representative movie of bulk cell movement; scale bar, 10 µm for the movie showing single-cell dynamics. Frame rate, 10 frames per second.

**Video 5.** **In complex environments, MT depolymerization causes loss of coherence.** DCs, either untreated (control) or treated with the indicated compounds (nocodazole or double treatment with Y27632 and nocodazole) were recorded in 60-s intervals while migrating within a path choice assay toward a soluble CCL19 gradient. Note that under all conditions, cells insert multiple protrusions into different channels when reaching the junction point (black arrowheads). Red arrowheads highlight rupturing events and loss of cellular coherence (only observed in drug-treated cells). Time in [min:s]. Scale bar, 10 µm. Frame rate, 10 frames per second.

We next tested the molecular link between MT dynamics and cellular retraction. As previously demonstrated in other cell types, nocodazole treatment triggered a global increase of RhoA activity and myosin light chain (MLC) phosphorylation (Liu et al., 1998; Takesono et al., 2010; Fig. S2, d and e), and pharmacological inhibition of the effector kinase Rho-associated protein kinase (ROCK) by Y27632 reverted this effect. Accordingly, in linear channels, nocodazole-induced directional switching was reverted by additional ROCK inhibition (Fig. 3, f and g; and Video 6). Together, these data indicated that directional switching is caused by a hyperactive contractile module that is destabilized in its localization.

**Video 6.** **Cell coherence is maintained in nocodazole-treated DCs migrating in channels.** Mature DCs migrating along a soluble CCL19 gradient within a straight microchannel. Montage shows separately acquired bright-field movies of non-treated, nocodazole-treated, and Y27632 and nocodazole double-treated cells. Images were acquired in 20-s intervals for 5 h. Note the directional oscillations of nocodazole only–treated cells. Time in [min:s]. Scale bar, 10 µm. Frame rate, 10 frames per second.

In contrast to linear channels, ROCK inhibition failed to rescue cell integrity and locomotion when MTs were depleted upon migration in complex environments (Fig. 3, i–l; and Video 5). Here, contractility is rate-limiting for locomotion, and ROCK inhibition alone caused the cells to entangle (Fig. 3 m). Together, these data add evidence that MTs act upstream of the contractile module and that actomyosin contractility is locally coordinated by MT depolymerization, which effectively coordinates competing protrusions when cells migrate through complex environments.

### The RhoA GEF Lfc associates with MTs and accumulates at sites of retraction

One established molecular link between MT depolymerization and actomyosin contraction is the MT-regulated RhoA guanine nucleotide exchange factor (GEF) Lfc, the murine homologue of GEF-H1. When Lfc is sequestered to MTs, it is locked in its inactive state, and only upon release from MTs, it is targeted to membrane-associated sites where it becomes active and triggers actomyosin contraction via RhoA and its effectors ROCK and MLC kinase (Krendel et al., 2002; Ren et al., 1998; Azoitei et al., 2019).

**Figure 4.**
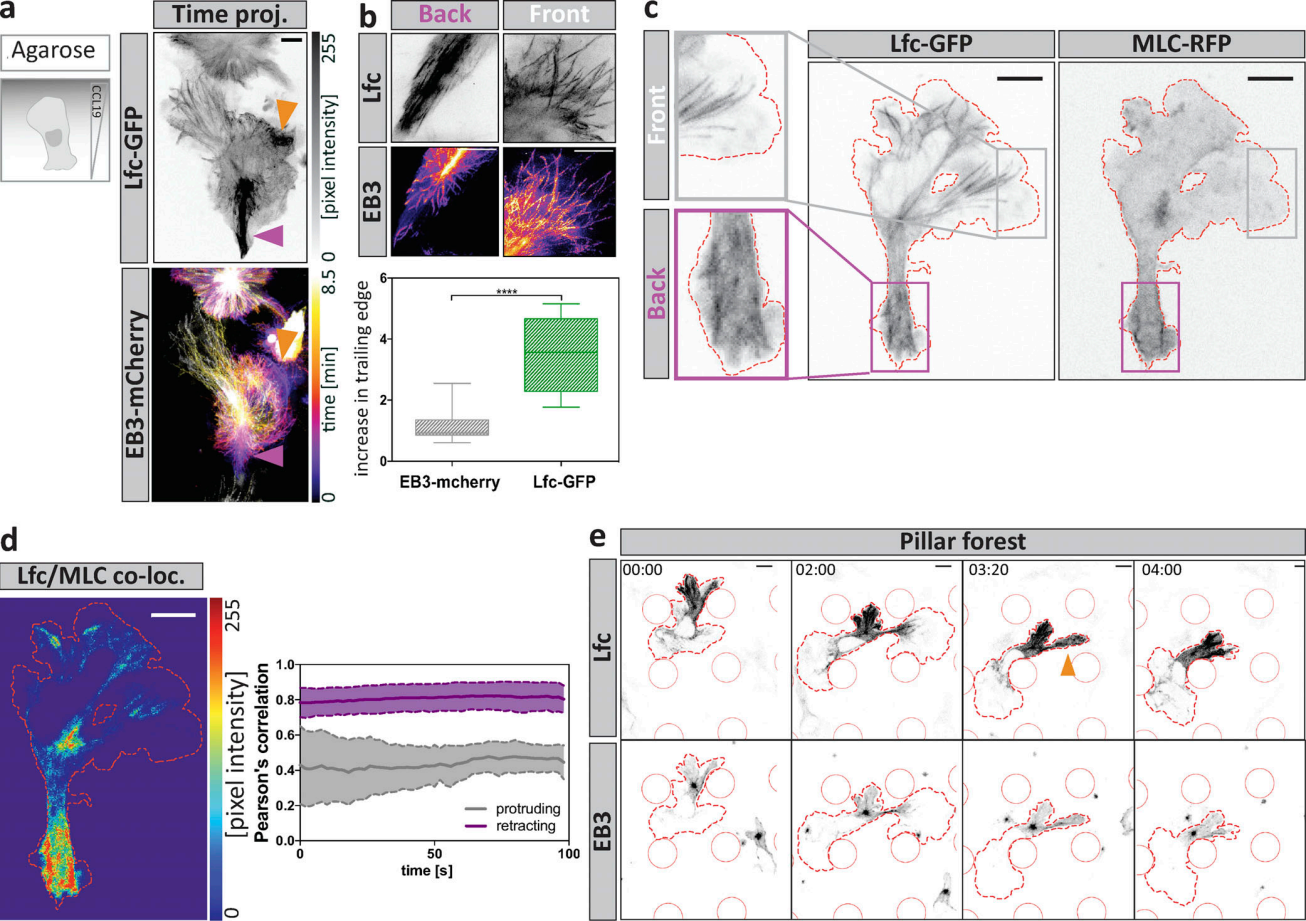
**The RhoA GEF Lfc associates with MTs and accumulates at sites of retraction. (a)** Polarized distribution of Lfc-GFP during DC migration. Maximum intensity time projection (proj.) of a double-fluorescent reporter cell expressing Lfc-GFP and EB3-mCherry over 8.5 min. Diffuse Lfc-GFP accumulation is highlighted in the trailing edge (purple arrowheads) and in retracting protrusions (orange arrowheads). Scale bar, 10 µm. **(b)** Enrichment of non-filamentous Lfc-GFP or EB3-mCherry signal in the rear versus the front of migrating cells. Maximum intensity time projection over 100 s. Scale bar, 5 µm. Lower panel: Relative enrichment of non-filamentous fluorescence signal intensities of Lfc-GFP and EB3-mCherry in the rear versus the front of *n* = 16 cells from *N* = 3 experiments. Boxes extend from 25th to 75th percentiles. Whiskers span minimum to maximum values. Annotation above columns indicates results of unpaired Student’s *t* test; ****, P ≤ 0.0001. **(c)** Differential localization of Lfc-GFP and MLC-RFP in protrusive (front, gray box) or contractile (back, purple box) area. Scale bar, 10 µm. **(d)** Co-localization (co-loc.) between Lfc-GFP and MLC-RFP; hot colors indicate strong co-localization, and cold colors specify exclusion. Right graph shows the correlation of co-localization over time. Boxed regions in c indicate exemplary regions used for the analysis of *n* = 8 cells ± SD. Co-localization was determined separately in actively protruding (gray box) and retracting (purple box) areas. **(e)** Distribution of Lfc-GFP and EB3-mCherry during migration within a pillar array. Time course of protrusion formation and protrusion retraction of a migrating fluorescent reporter cell. Dashed red line indicates cell outline; solid red line, individual pillars. Orange arrowhead indicates Lfc-GFP accumulation during protrusion retraction. Scale bar, 5 µm. See also Video 7.

To determine whether Lfc might be involved in MT-mediated cellular retraction events during amoeboid migration, we first mapped Lfc distribution by visualizing an Lfc-GFP fusion protein. Immunofluorescence of α-tubulin in Lfc-GFP–expressing cells confirmed the localization of Lfc-GFP to MT filaments (Krendel et al., 2002; Fig. S2 f), with highest signal intensities in trailing edge areas (Fig. 4, a and b; purple arrowhead in a). Besides its clear filamentous appearance across the cell, Lfc-GFP accumulated as a diffuse patch in trailing edges and in retracting protrusions (Fig. 4 a, orange arrowhead; Fig. S2 g; and Video 7). Treatment with nocodazole globally changed Lfc distribution from filamentous to diffuse (Fig. S2 h).

**Video 7.** **Polarized Lfc-GFP distribution precedes retraction of explorative protrusions.** A DC expressing Lfc-GFP and EB3-mCherry was acquired while migrating under agarose (first part) or within a 3D pillar array (last part) toward a soluble CCL19 gradient in 2-s intervals on an inverted spinning-disk microscope (inverted signal). Purple arrowheads denote persistent diffuse trailing edge Lfc-GFP signal. Orange arrowheads highlight protrusion-retraction accompanied by a change of Lfc-GFP signal distribution. White and black arrowheads indicate filamentous Lfc-GFP signal distribution in protruding areas after repolarization. Time in [min:s]. Scale bar, 10 µm. Frame rate, 20 frames per second.

To test whether Lfc accumulates in actively retracting areas, we determined the spatiotemporal co-localization of Lfc and MLC by imaging double-transfected cells migrating under agarose. Time-course analysis revealed that both proteins are strongly polarized in trailing edge regions and at the cell center in close proximity to the nucleus during phases of cell body translocation (Fig. 4 c). Correlation coefficients of Lfc and MLC in retracting areas were positive over time, indicating that locally increased Lfc levels are paralleled by increased MLC signal intensities in these regions (Fig. 4 d). This pattern was particularly prominent when DCs migrated through pillar forests (Fig. 4 e). Here, Lfc-GFP transiently accumulated in peripheral explorative protrusions and at the trailing edge (Fig. 4 e and Video 7). Together, these data show that Lfc associates with MTs and locally accumulates, together with MLC, at sites of retraction.

### Lfc promotes MLC localization at the cell periphery

**Figure S3.**
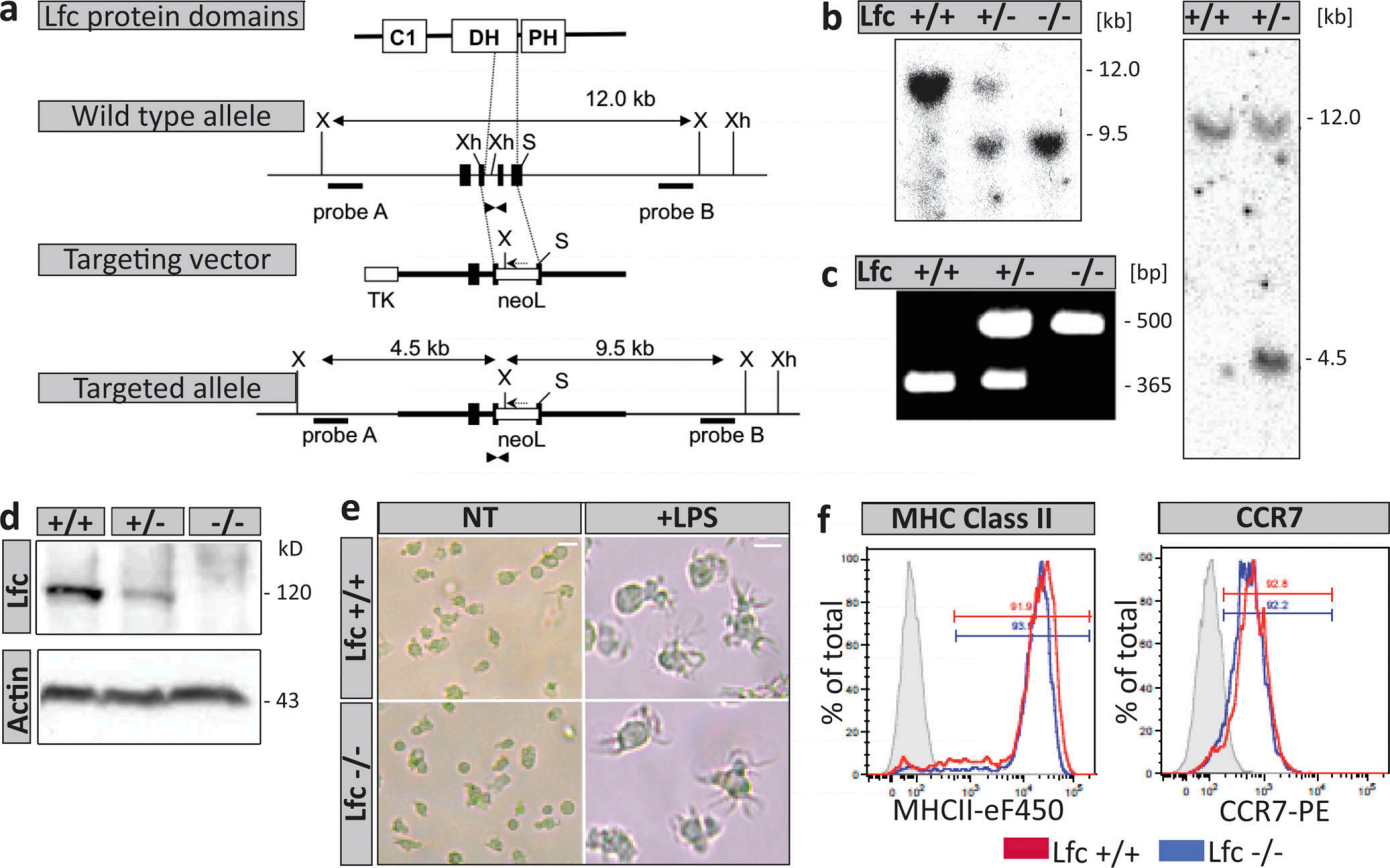
****Disruption of the Lfc gene locus**.** The targeting vector was constructed by substituting genomic DNA corresponding to the end of the Dbl homology (DH) domain and the interface between DH and pleckstrin homology (PH) domains with a LoxP-*Neo*-LoxP cassette in order to generate a null mutation. **(a)** Integration of the Lfc targeting vector into the genomic locus. Black boxes represent exons. The neo-LoxP (neoL) cassette was cloned in reverse orientation into two exons (one coding for the DH domain, the other for the DH/PH interface), replacing a *SmaI-XhoI* segment. Locations of primers used for PCR are indicated with triangles. Probes A and B were used for Southern blot detection of short and long arms, respectively. S, *Sma*I; Xh, *Xho*I; X, *Xba*I; DH, Dbl homology; PH, Pleckstrin homology. **(b)** Southern blot analysis. Left panel: Genomic DNA from Lfc^+/+^, Lfc^+/−^, and Lfc*^−/−^* mice was digested with *Xba*I and hybridized with probes B. Right panel: Genomic DNA from Lfc^+/+^ and Lfc^+/−^ embryonic stem cells was hybridized with probe A. **(c)** PCR analysis of tail DNA from Lfc^+/+^, Lfc^+/−^, and Lfc*^−/−^* mice. Locations of primers used for PCR are indicated with triangles in panel a. **(d)** Immunoblot analysis of total thymus cell lysates probed for Lfc protein content. **(e)** Cell morphologies of immature (NT) and mature (+LPS) Lfc wild type (upper-lane) and Lfc-deficient (lower-lane) littermate DCs. Note the presence of multiple veils in both LPS-treated samples. Scale bar, 10 µm. **(f)** DC differentiation markers (MHC-II and CCR7) of Lfc^+/+^ (blue line) and Lfc*^−/−^* (red line) littermate DCs compared with unstained cells (gray peak). eF450, eFlour 450; PE, Phycoerythrin.

**Figure 5.**
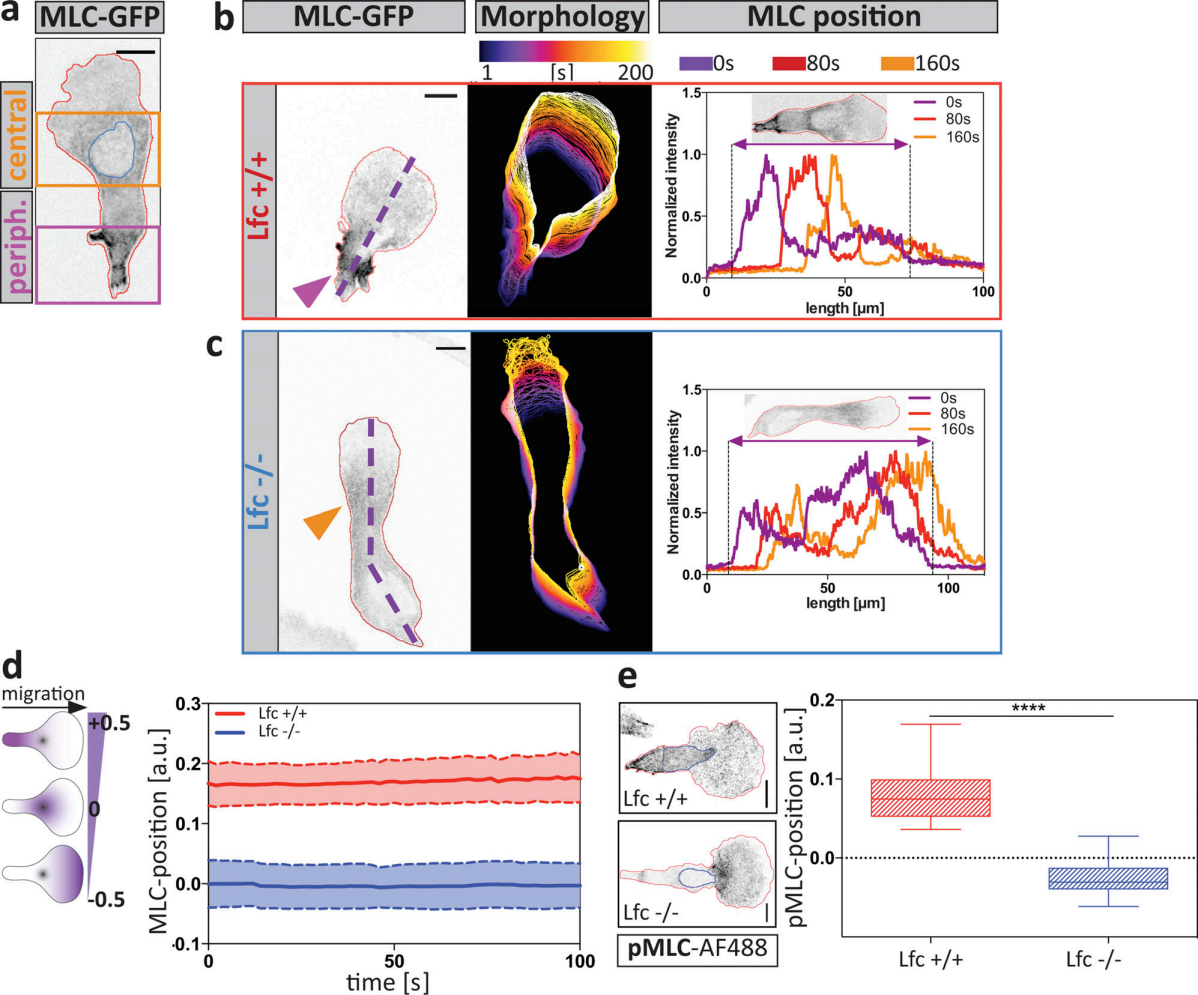
**Lfc specifies MLC localization at the cell periphery. (a)** An MLC-GFP–expressing DC migrating under agarose along a soluble CCL19 gradient. Central (orange box) and peripheral (periph.; purple box) MLC accumulation is outlined. The blue line indicates the position of the nucleus. The red line outlines cell shape. Scale bar, 10 µm. **(b and c)** MLC accumulation during migration under agarose in wild type (b) or Lfc^−/−^ (c) cells. Scale bar, 10 µm. Middle panels indicate cell shapes over time. Right panels indicate mean MLC fluorescence distribution along the anterior-posterior polarization axis (dashed line) in 80-s intervals. Arrowheads indicate peripheral (purple) and central (orange) MLC accumulation. **(d)** Localization of MLC accumulation during directed migration of Lfc^+/+^ (red) and Lfc^−/−^ (blue) DCs. To account for differences in cell length, the distance between cell center and MLC accumulation was normalized to cell length. Graph shows the distance over time of *n* = 7 migratory cells per condition ± SD. See also Video 8. **(e)** Left panel: Localization of endogenous phospho-MLC(S19) (pMLC) in fixed migratory DCs. The blue line indicates the position of the nucleus. The red line outlines cell shape. Right panel indicates the position of MLC accumulation relative to cell length of *n* = 16 cells per condition from *N* = 4 experiments. Boxes extend from 25th to 75th percentiles. Whiskers span minimum to maximum values. Annotation above columns indicates results of unpaired Student’s *t* test; ****, P ≤ 0.0001. Scale bar, 10 µm.

**Figure S4.**
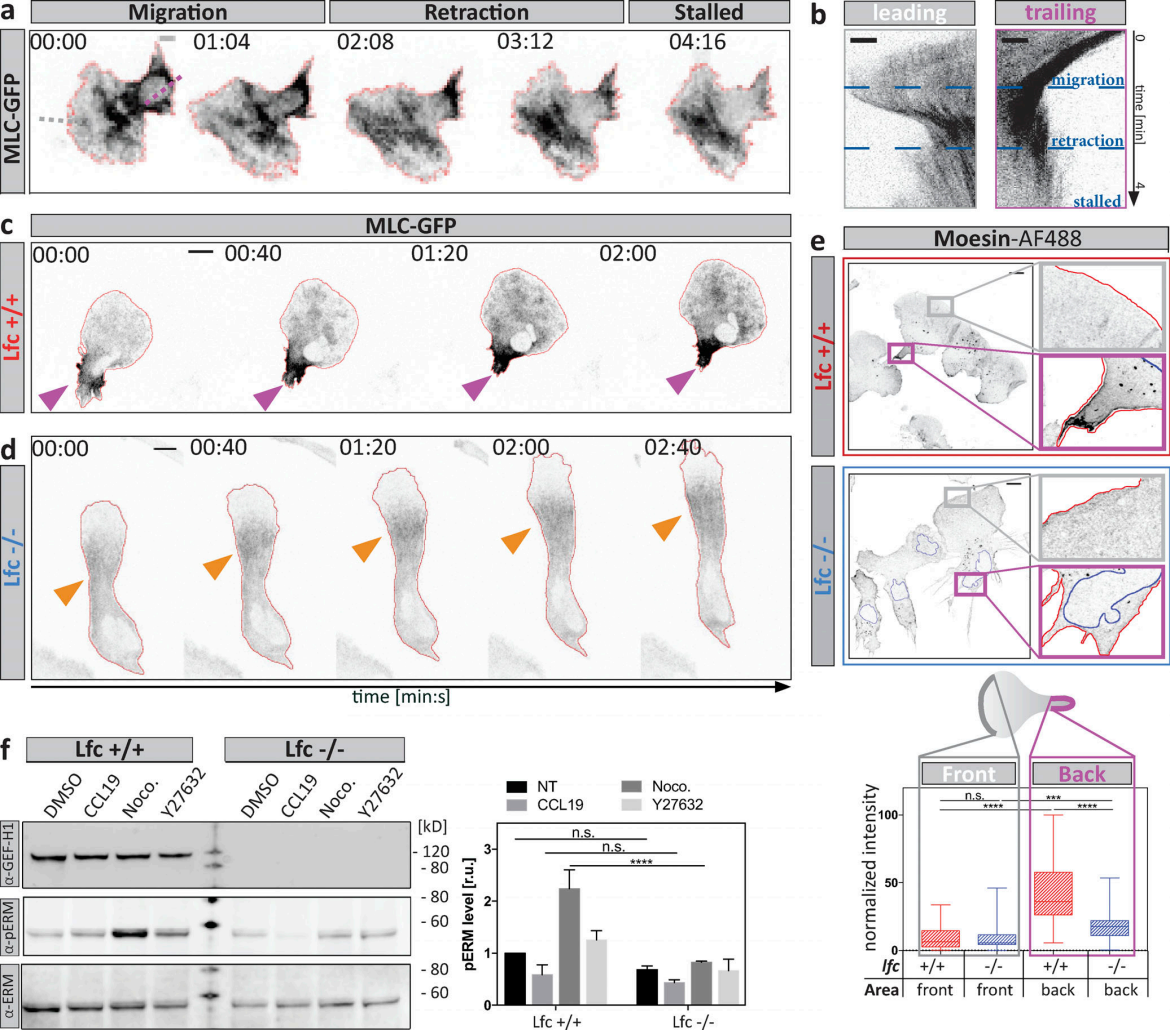
**Aberrant spatiotemporal MLC accumulation and moesin localization in Lfc***^−/−^*** DCs. (a)** Time-lapse montage of an MLC-GFP–expressing DC migrating under agarose toward a soluble CCL19 gradient. A cycle of migration, retraction, and pausing is shown. Scale bar, 10 µm. Dotted lines indicate positions further analyzed by kymographs in b. **(b)** Leading edge kymograph was derived from gray dotted line in leading edge region of panel a. Trailing edge kymograph was derived from purple dotted line in trailing edge region of panel a. Note the absence of MLC accumulation in leading edge areas and the presence of trailing edge MLC accumulation during the migration. Scale bar, 5 µm. **(c and d)** Time-lapse sequence showing spatiotemporal MLC accumulation of Lfc^+/+^ (c) and Lfc*^−/−^* (d) DCs. Purple arrowheads highlight the trailing edge MLC accumulation, and orange arrowheads indicate central MLC accumulation. Scale bars, 10 µm. **(e)** Quantitative morphometry of moesin in fixed migratory Lfc^+/+^ (red) and Lfc*^−/−^* (blue) DCs. Lower panel: Quantification of fluorescence intensity in the leading versus trailing edge regions of Lfc^+/+^ (red) and Lfc*^−/−^* (blue) DCs of *n* = 55 cells per condition from *N* = 3 experiments. Boxes extend from 25th to 75th percentiles. Whiskers span minimum to maximum values. ***, P ≤ 0.001; ****, P ≤ 0.0001. Scale bars, 10 µm. **(f)** Protein levels of phospho-ERM (pERM) in Lfc^+/+^ and Lfc*^−/−^* DCs assessed by Western blot analysis. Right panel: Quantification of pERM levels upon treatment with DMSO, CCL19, nocodazole (Noco.), or Y27632. Mean fluorescence intensity of pERM signal was normalized to total ERM signal and shown as fold increase relative to Lfc^+/+^ DMSO control ± SD of *N* = 3 experiments. Annotation above columns indicates results of two-way ANOVA; ****, P ≤ 0.0001. n.s., not significant; r.u., relative units; NT, non-treated; ERM, Ezrin/Radixin/Moesin.

To functionally test whether Lfc is involved in coordinating multiple protrusions, we knocked out *Arhgef2*, the Lfc-encoding gene in mice (Fig. S3, a–d) and generated phenotypically normal DCs from hematopoietic precursor cells (Fig. S3, e and f). We first asked how the Lfc pathway affects the localization of the contractile module and dynamically visualized MLC localization in Lfc^−/−^ and wild type DCs migrating under agarose toward CCL19 gradients. While MLC was largely excluded from the leading lamellipodium, two distinct pools were detectable in the cell body of wild type cells: one at the peripheral trailing edge and one in the cell center, at the base of the lamellipodium and around the nucleus (Fig. 5 a; and Fig. S4, a and b). Quantification of the distance between the center of mass and center of MLC signal showed that migrating Lfc^−/−^ cells completely lost MLC polarization at the cell periphery but maintained MLC in the cell center (Fig. 5, b–d; Fig. S4, c and d; and Video 8). The same distribution pattern was obtained by determining the localization of the active form of MLC (phospho-MLC; Fig. 5 e) and its effector protein moesin (Fig. S4, e and f) in fixed samples. This demonstrates that, in correspondence with the Lfc localization data, Lfc deficiency led to a loss of MLC localization in peripheral parts of the cell while central MLC localization was not affected.

**Video 8.** **Lfc mediates myosin localization at the trailing edge.** Combined movies of MLC-GFP–expressing Lfc^+/+^ DCs (left panel) and Lfc^−/−^ DCs (right panel) migrating under agarose along a soluble CCL19 gradient, acquired in 2-s intervals on an inverted spinning-disk microscope (inverted signal). Magenta arrowhead indicates trailing edge MLC accumulation, which is absent in Lfc^−/−^ cells. Orange arrowhead highlights central MLC accumulation. Time in [min:s]. Scale bar, 10 µm. Frame rate, 10 frames per second.

### Loss of Lfc causes DC entanglement

**Figure 6.**
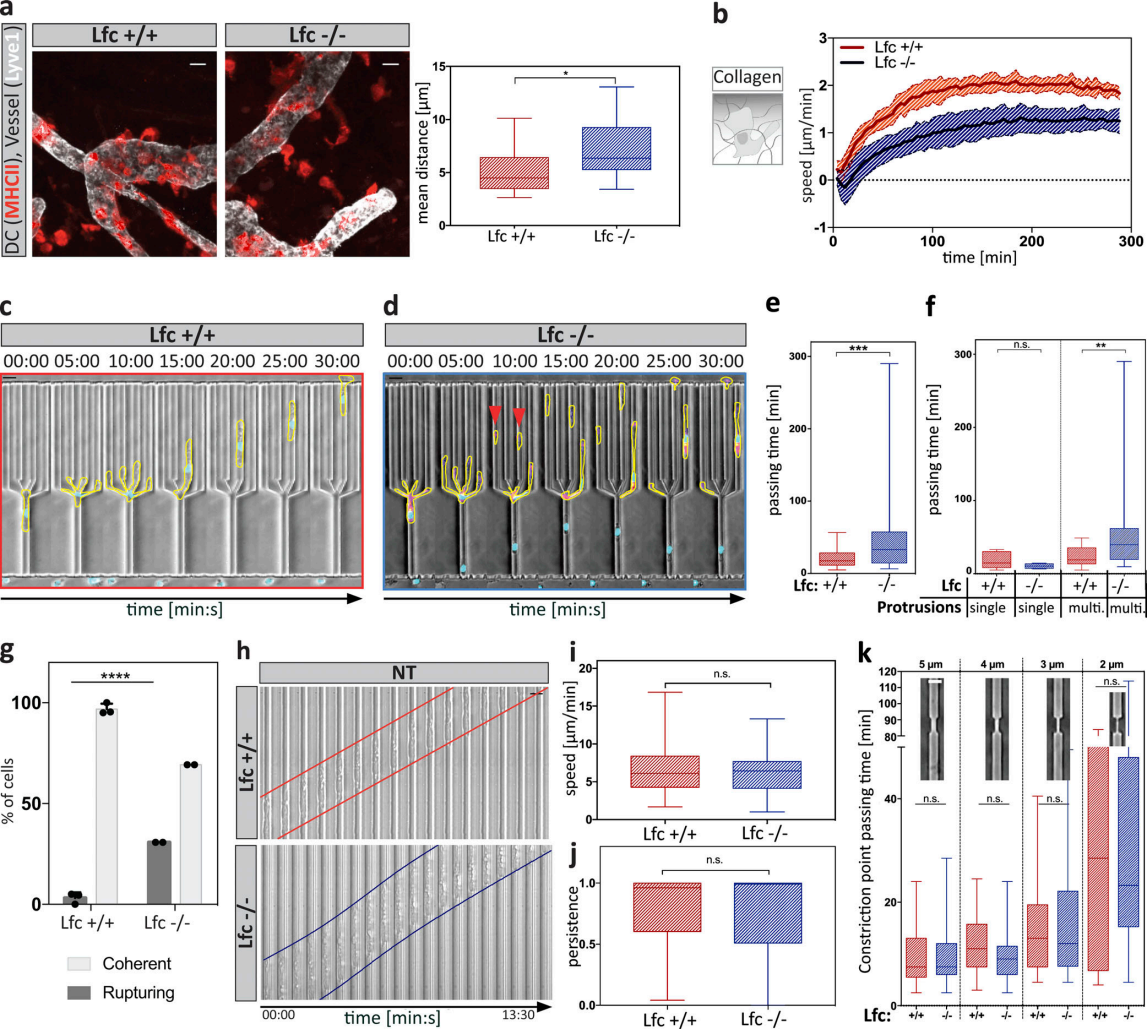
**Loss of Lfc causes DC entanglement. (a)** In situ migration of exogenous DCs on a mouse ear sheet. Lymphatic vessels were stained for Lyve-1 and DCs with TAMRA. Right panel iindicates the mean distance of cells from lymphatic vessels. Per experiment, two mouse ears with two fields of view were analyzed from *n* = 4 experiments. Boxes extend from 25th to 75th percentiles. Whiskers span minimum to maximum values. Annotation above columns indicates results of unpaired Student’s *t* test; *, P ≤ 0.05. Scale bar, 100 µm. **(b)** Automated analysis of y-directed migration speed within a collagen network along a soluble CCL19 gradient. Plot shows mean population migration velocities over time ± SD from *N* = 7 experiments. **(c)** Time-lapse sequence of a wild type littermate control cell migrating within a path choice device. Scale bar, 10 µm. **(d)** Time-lapse sequence of an Lfc^−/−^ cell migrating within a path choice device. Red arrowheads denote multiple rupturing events of an individual cell. Scale bar, 10 µm. **(e)** Junction point passing times of Lfc^+/+^ (*n* = 79 cells from *N* = 3 experiments) and Lfc^−/−^ (*n* = 49 cells from *N* = 2 experiments) DCs. Boxes extend from 25th to 75th percentiles. Whiskers span minimum to maximum values. Annotation above columns indicates results of unpaired Mann-Whitney test; ***, P ≤ 0.001. See also Video 9. **(f)** Junction point passing times depending on presence of single non-competing or multiple (multi.) competing protrusions per cell of Lfc^+/+^ (*n* = 37 cells from *N* = 3 experiments) and Lfc^−/−^ (*n* = 46 cells from *N* = 2 experiments) DCs. Boxes extend from 25th to 75th percentiles. Whiskers span minimum to maximum values. Annotation above columns indicates results of Kruskal-Wallis with Dunn’s test; **, P ≤ 0.01. **(g)** Frequency of cell rupturing events during migration within path choice device of Lfc^+/+^ (*n* = 79 cells ± SD from *N* = 3 experiments) and Lfc^−/−^ (*n* = 52 cells ± SD from *N* = 2 experiments) DCs. Annotation above columns indicates results of two-way ANOVA with Sidak’s test; ****, P ≤ 0.0001. **(h)** Migration of DCs within straight microchannels. Cell edges are indicated in red (Lfc^+/+^) and blue (Lfc^-/-^). NT, non-treated. Scale bar, 10μm. **(i)** Migration speed of Lfc^+/+^ and Lfc^−/−^ DCs within straight microchannels of *n* = minimum of 80 cells per condition from *N* = 5 experiments. Boxes extend from 25th to 75th percentiles. Whiskers span minimum to maximum values. Annotation above columns indicates results of one-way ANOVA. **(j)** Migratory persistence of Lfc^+/+^ and Lfc^−/−^ DCs within straight microchannels of *n* = minimum of 80 cells per condition from *N* = 5 experiments. Boxes extend from 25th to 75th percentiles. Whiskers span minimum to maximum values. Annotation above columns indicates results of Kruskal-Wallis with Dunn’s test. **(k)** Single constriction passing times of Lfc^+/+^ (*n* = 114 cells from *N* = 3 experiments) and Lfc^−/−^ (*n* = 195 cells from *N* = 3 experiments) DCs. Boxes extend from 25th to 75th percentiles. Whiskers span minimum to maximum values. Annotation above columns indicates results of Kruskal-Wallis with Dunn’s test. n.s., not significant.

**Figure S5.**
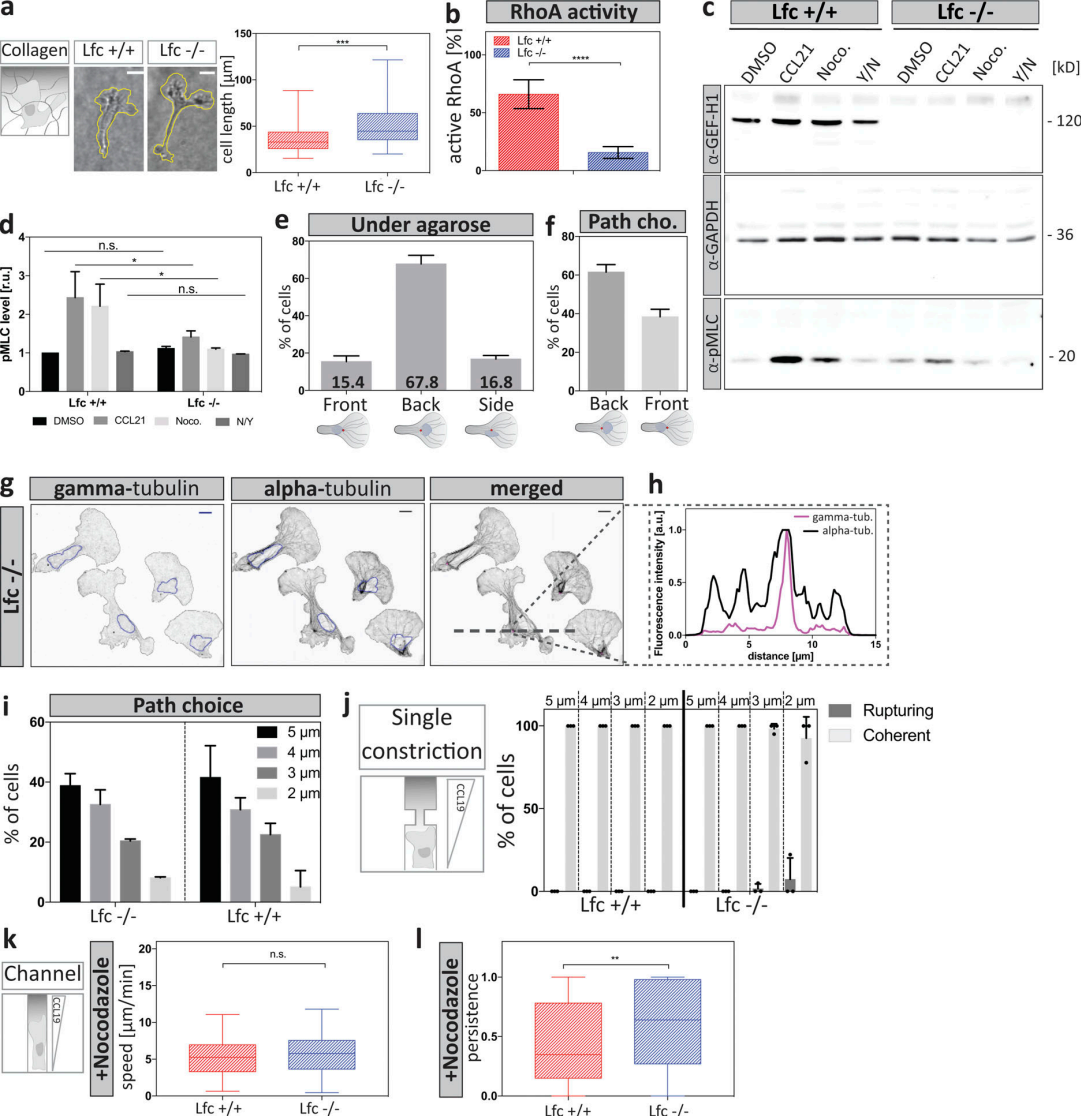
**Lfc*^−/−^* DCs exhibit reduced contractile responses. (a)** Left: Cell outlines of Lfc^+/+^ (left) and Lfc*^−/−^* (right) DCs migrating within a collagen network along a soluble CCL19 gradient. Scale bar, 10 µm. Right: Graph shows the lengths of cells migrating within a collagen network of *n* = 85 individual cells per condition from *N* = 4 experiments. Boxes extend from 25th to 75th percentiles. Whiskers span minimum to maximum values. Annotation above columns indicates results of unpaired Student’s *t* test; ***, P ≤ 0.001. **(b)** Levels of active RhoA of Lfc^+/+^ and Lfc*^−/−^* cells were determined by luminometry showing the mean intensities ± SD from *N* = 3 experiments. Annotation above columns indicates results of unpaired Student’s *t* test; ****, P ≤ 0.0001. **(c)** Levels of MLC phosphorylation (pMLC) in Lfc^+/+^ and Lfc*^−/−^* DCs assessed by Western blot analysis. Cells were treated with the indicated compounds (DMSO, CCL21, nocodazole [Noco.], or Y27632 together with nocodazole [Y/N]). **(d)** Mean fluorescence intensity of phospho-MLC was normalized to GAPDH signal and shown as fold increase relative to DMSO control ± SD. Blots are representative of *N* = 3 experiments. Annotation above columns indicates results of two-way ANOVA; *, P ≤ 0.1. r.u., relative units. **(e)** Centrosome localization relative to the nucleus in Lfc*^−/−^* DCs migrating under agarose assessed by α- and γ-tubulin costaining (*n* = 117 cells from *N* = 2 experiments ±SD). **(f)** Centrosome position relative to the nucleus of Lfc*^−/−^* DCs migrating within a path choice assay. Shown are mean frequencies of *n* = 49 cells from *N* = 2 experiments ±SD. cho., choice. **(g)** MT nucleation from centrosomal origin as determined by α- and γ-tubulin costaining. Blue line indicates the position of the nucleus. Scale bars, 10 µm. **(h)** Intensity line profiles across the highest γ-tubulin (tub.) signal along the left-right axis (dashed line in g). The purple line indicates γ-tubulin signal intensity. The black line indicates α-tubulin signal distribution. **(i) **Path choice preference of Lfc^+/+^ and Lfc*^−/−^* DCs migrating within a path choice assay. Shown are mean frequencies of Lfc*^−/−^* (*n* = 49 cells from *N *= 2 experiments) and Lfc^+/+^ (*n* = 79 cells from *N* = 3 experiments) DCs ±SD. **(j)** Frequency of cell rupturing events of Lfc^+/+^ (*n* = 73 cells from *N* = 3 experiments) and Lfc*^−/−^* (*n* = 128 cells from *N* = 3 experiments) DCs while migrating within single constriction–containing microchannels ±SD. **(k)** Migration speed of nocodazole-treated cells within straight microchannels of *n* = minimum of 80 cells per condition from *N* = 5 experiments. Boxes extend from 25th to 75th percentiles. Whiskers span minimum to maximum values. Annotation above columns indicates results of one-way ANOVA with Tukey’s test. **(l)** Migratory persistence of nocodazole-treated Lfc^+/+^ and Lfc*^−/−^* DCs within straight microchannels of *n* = minimum of 80 cells per condition from *N* = 5 experiments. Boxes extend from 25th to 75th percentiles. Whiskers span minimum to maximum values. Annotation above columns indicates results of Kruskal-Wallis with Dunn’s test; **, P ≤ 0.01. n.s., not significant.

To address how defective subcellular MLC localization translates into function, we next measured the migratory capacity of Lfc^−/−^ DCs under physiological conditions. In situ migration in explanted ear sheets showed that Lfc^−/−^ cells reached the lymphatic vessels later than control cells (Fig. 6 a) and also that chemotaxis of Lfc^−/−^ DCs in collagen gels was substantially impaired (Fig. 6 b). When we measured cell lengths in 3D collagen gels, Lfc^−/−^ DCs were significantly elongated compared with control cells, indicating retraction defects (Fig. S5 a).

To more directly address the potential retraction defect of Lfc^−/−^ DCs, we turned back to the microfluidic devices and placed mature DCs into bifurcating channels (Fig. 6, c and d). In line with the finding that Lfc mediates between MTs and myosin II (Fig. S5, b–d), Lfc^−/−^ DCs showed increased passage times due to defective retraction of supernumerary protrusions (Fig. 6, e and f). Lfc^−/−^ DCs did not show obvious differences in MT organization (Fig. S5, e–h) or path choice preference (Fig. S5 i). Notably, similar to nocodazole-treated cells, Lfc^−/−^ DCs advanced through more than one channel (Fig. 6 d), resulting in auto-fragmentation into migratory cytoplasts in more than 25% of the cells (Fig. 6 g and Video 9). When cells migrated in straight channels and even when confronted with single constrictions, Lfc^−/−^ cells passed with the same speed and efficiency as wild type cells (Fig. 6, h–k; and Fig. S5, j–l). This demonstrates that neither locomotion nor passage through constrictions was perturbed in the absence of Lfc but rather the coordination of competing protrusions. These data indicate that in complex 3D geometries, where the cell has to choose between different paths, MTs—via Lfc and myosin II—mediate the retraction of entangled protrusions.

**Video 9.** **Microtubules mediate the retraction of supernumerary protrusions via Lfc.** Lfc^+/+^ and Lfc^−/−^ DCs were recorded while migrating within a path choice assay toward a soluble CCL19 gradient in 30-s intervals. Note that both genotypes insert multiple protrusions into different channels when reaching the junction point (black arrowheads). Red arrowheads highlight rupturing events and loss of cellular coherence (only observed in Lfc^−/−^ cells). Time in [min:s]. Scale bar, 10 µm. Frame rate, 10 frames per second.

### Loss of Lfc causes retraction failure when DCs migrate in an adhesive mode

**Figure 7.**
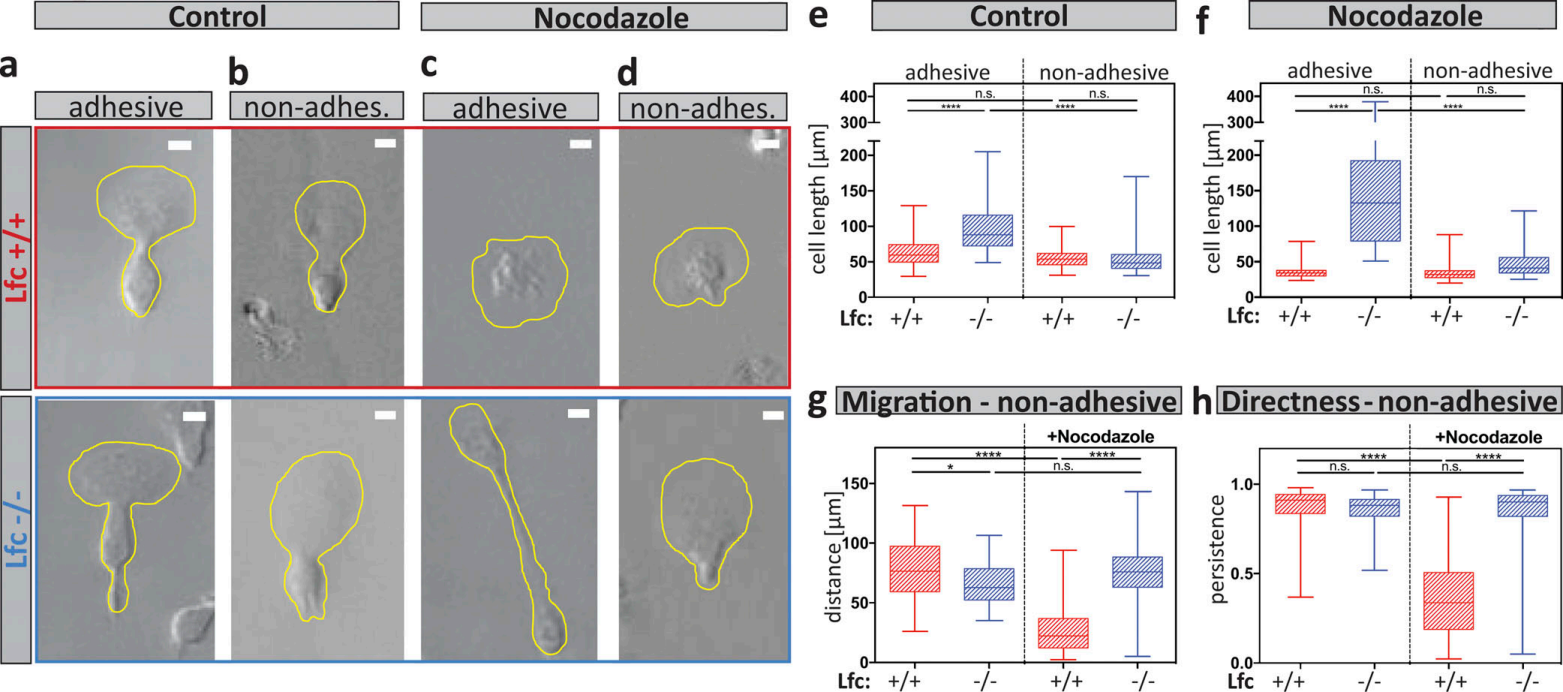
**Lfc regulates microtubule-mediated adhesion resolution.** **(a–d)** Cell shape outlines of non-treated control cells migrating under agarose under adhesive (a) or non-adhesive (PEG-coated; b) conditions. Cell shape outlines of nocodazole-treated cells migrating under agarose under adhesive (c) or non-adhesive (PEG-coated; d) conditions. Upper panels show littermate control wild-type cells. Lower panels show Lfc^−/−^ cells. Scale bar, 10 µm. See also Video 10. **(e)** Cell lengths of non-treated control cells migrating under adhesive and non-adhesive conditions (*n* = minimum of 80 cells per condition from *N* = 5 experiments). Boxes extend from 25th to 75th percentiles. Whiskers span minimum to maximum values. Annotation above columns indicates results of Kruskal-Wallis with Dunn’s test; ****, P ≤ 0.0001. **(f)** Cell lengths of nocodazole-treated cells migrating under adhesive and non-adhesive conditions (*n* = minimum of 80 cells per condition from *N* = 5 experiments). Boxes extend from 25th to 75th percentiles. Whiskers span minimum to maximum values. Annotation above columns indicates results of Kruskal-Wallis with Dunn’s test; ****, P ≤ 0.0001. **(g)** Migration distance of Lfc^+/+^ and Lfc^−/−^ DCs migrating under agarose under non-adhesive (PEG-coated) conditions of *n* = minimum of 80 cells per condition from *N* = 5 experiments. Cells were either non-treated or treated with nocodazole. Boxes extend from 25th to 75th percentiles. Whiskers span minimum to maximum values. Annotation above columns indicates results of one-way ANOVA; *, P ≤ 0.05; ****, P ≤ 0.0001. **(h)** Persistence of Lfc^+/+^ and Lfc^−/−^ DCs migrating under agarose under non-adhesive conditions (PEG-coated). Cells were either non-treated or nocodazole-treated (*n* = minimum of 80 cells per condition from *N* = 5 experiments). Boxes extend from 25th to 75th percentiles. Whiskers span minimum to maximum values. Annotation above columns indicates results of Kruskal-Wallis with Dunn’s test; ****, P ≤ 0.0001. non-adhes., non-adhesive; n.s., not significant.

**Figure 8.**
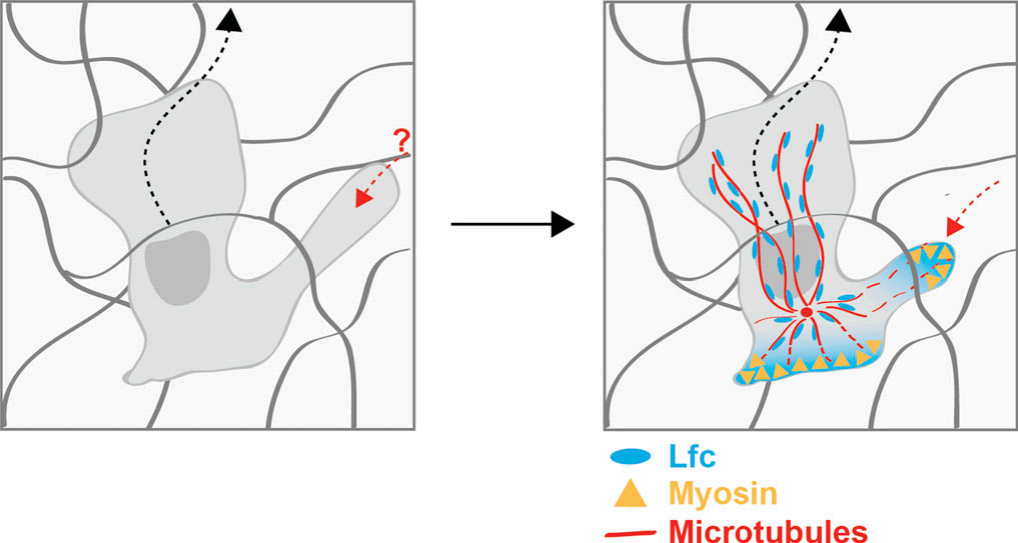
**Schematic illustration of MT-mediated pathfinding in complex 3D environments.** Left panel: DCs extend multiple protrusion when navigating through the interstitium. In order to maintain cell coherence, side protrusions have to be retracted. The mechanistic basis of coordinating multiple protrusions in complex 3D environments is not understood (red question mark). Right panel: To coordinate multiple protrusions and avoid cell entanglement, MTs depolymerize (dashed red lines) and release Lfc, which leads to actomyosin activation and retraction of competing protrusions (dashed red arrow).

In cells that employ an amoeboid mode of migration, defective retraction cannot only stall locomotion by entanglement, as we showed in microfluidic channels, but it may also lead to failed disassembly of integrin adhesion sites. We therefore tested the role of adhesion resolution in under-agarose assays, where, depending on the surface conditions, DCs can flexibly shift between adhesion-dependent and adhesion-independent locomotion (Renkawitz et al., 2009). Under adhesive conditions, Lfc^−/−^ DCs were elongated compared with wild type cells (Fig. 7 a), and this elongation was lost when the migratory substrate at the bottom was passivated with polyethylene glycol (PEG; Fig. 7, b and e). When cells on adhesive surfaces were treated with nocodazole, wild type cells shortened as expected due to hypercontractility (Fig. 7 c). Notably, Lfc^−/−^ DCs elongated even more upon treatment with nocodazole (Fig. 7 c; lower panel), indicating that elimination of Lfc-mediated hypercontractility unmasked additional modes of MT-mediated length control. Elongation of Lfc^−/−^ cells by nocodazole was also largely absent on PEG-coated surfaces (Fig. 7, d and f; and Video 10). Importantly, not only morphological but also migratory parameters were restored on passivated surfaces (Fig. 7, g and h). Together, these data demonstrate that whenever DCs migrate in an adhesion-mediated manner, MTs control de-adhesion, and this is partially mediated via Lfc and myosin II. We conclude that MT depolymerization in peripheral regions of migrating DCs locally triggers actomyosin-mediated retraction via the RhoA GEF Lfc. Thus, MTs coordinate protrusion-retraction dynamics and prevent the cell from getting too long or ramified (Fig. 8).

**Video 10.** **Lfc regulates MT-mediated adhesion resolution.** Nocodazole-treated Lfc^+/+^ and Lfc^−/−^ DCs were acquired while migrating under agarose toward a soluble CCL19 gradient in 20-s intervals on an inverted cell culture microscope. Left panels show nocodazole-treated cells during adhesive migration. Note the loss of directionality in Lfc^+/+^ DCs and the pronounced elongation of Lfc^−/−^ DCs. Right panels show nocodazole effects during adhesion-independent migration on PEG-coated coverslips. Note the persistent loss of directionality in Lfc^+/+^ DCs but the restored cell lengths of Lfc^−/−^ DCs. Time in [min:s]. Scale bar, 100 µm. Frame rate, 20 frames per second.

## Discussion

Here, we report that MT depolymerization in peripheral regions of migrating DCs locally triggers actomyosin-mediated retraction via the RhoA GEF Lfc. Based on our findings, we propose a model of cellular proprioception that can act independently of the two prevalent modes that involve actin stress fibers and communication by membrane tension: in our model, dynamic MTs take the role of the shape sensor, and the state of the MT system then signals to actin dynamics. This pathway may be particularly relevant for leukocytes, as they do not develop stress fibers due to low adhesive forces and are often too large and ramified (such as DCs in 3D matrices) to allow equilibration of membrane tension across the cell body (Shi et al., 2018).

Although it is likely that multiple feedback loops signal between actin and MTs, we show that there is a strong causal link between local MT catastrophes and cellular retraction, with MTs acting upstream. This raises the key question of how MT stability is locally regulated in DCs. Among many possible inputs (adhesion, chemotactic signals, etc.), one simple option may be related to the fact that in leukocytes, the MTOC is the only site where substantial nucleation of MTs occurs. In complex environments (such as the pillar maze we devised), the MTOC of a DC moves on a remarkably straight path, while lateral protrusions constantly explore the environment (Fig. 1 b). Hence, the passage of the MTOC beyond an obstacle and through a gap is the decisive event determining the future trajectory of the cell. Upon passage of the MTOC, sheer geometry may determine that all but the leading protrusion are cut off from MT supply because MTs are too inflexible to find their way into curved, narrow, and ramified spaces. Consequently, we propose that MTs serve as an internal explorative system of the cell that informs actomyosin whenever a peripheral protrusion locates too distant from the centroid and thereby initiates its retraction.

Despite being therapeutically targeted, the role of MTs in leukocytes is poorly studied. In neutrophil granulocytes and T cells, it was shown that pharmacological MT depolymerization leads to enhanced cellular polarization, owing to a hypercontractility-induced symmetry break that triggers locomotion but at the same time impairs directional persistence and chemotactic prowess (Redd et al., 2006; Xu et al., 2005; Takesono et al., 2010; Yoo et al., 2012). Although this pharmacological effect might explain the efficacy of MT depolymerizing drugs such as Colchicine in the treatment of neutrophilic hyperinflammation, excessive hypercontractility overwrites any morphodynamic subtleties and leaves the question if MTs contribute to leukocyte navigation under physiological conditions. Our findings demonstrate that in DCs, this is indeed the case and that the MT-sequestered RhoA GEF Lfc is an important mediator between MT dynamics and actomyosin-driven retraction. Importantly, we show that DCs lacking both Lfc and MTs had even more severe cell shape defects than the ones lacking Lfc only. This demonstrates that Lfc and myosin II are not the only pathways and that MT depolymerization induces cell retraction via additional modes that remain to be identified.

## Materials and methods

### Mice

All mice used in this study were bred on a C57BL/6J background and maintained at the institutional animal facility in accordance with the Institute of Science and Technology Austria ethics commission and Austrian law for animal experimentation. Permission for all experimental procedures was granted and approved by the Austrian Federal Ministry of Education, Science and Research (identification code: BMWF-66.018/0005-II/3b/2012).

### Generation of Lfc^−/−^ mice

A cosmid containing the full genomic sequence of the gene that encodes Lfc (*Arhgef2*) was isolated from a 129-mouse genomic library with Lfc cDNA probes (106–630, 631–1,057, and 1,060–1,478 bp) amplified by RT-PCR. The genomic DNA region between base pairs 1193 and 1477, coding for amino acids 351–445 in the Dbl homology (DH) domain, and the DH/Pleckstrin homology domain interface were exchanged for a neomycin cassette flanked by LoxP sites. The targeting construct was linearized with *Not*I and electroporated into R1 embryonic stem cells. Homologous recombinants were selected in the presence of G418 (150 µg/ml) and gancyclovir (2 µM) and analyzed by Southern blotting. Positive embryonic stem cell clones were aggregated with eight cell-stage mouse embryos to generate chimeras. The resulting mice were genotyped by Southern blot and PCR. Primers (5′-CGG​GGA​TCC​ATT​CGG​TTG​TAA-3′ and 5′-AAG​CGG​CAT​GGA​GTT​CAG​GA-3′) amplified a 365-bp fragment specific for the wild type allele, whereas primers (5′-AGA​GTT​CTG​CAG​CCG​CCA​CAC​CA-3′) and (5′-GGT​GGG​GGT​GGG​GTG​GGA​TTA​GAT​A-3′) amplified a 500-bp fragment specific for the targeted allele. We refer to these mice as Lfc^−/−^ mice throughout the entire manuscript. Western blot analysis using an Lfc-specific antibody was performed to confirm that Lfc^−/−^ mice had no expression of Lfc protein. Mice were backcrossed to C57BL/6 background for more than 12 generations. Dendritic cells were generated from bone marrow isolated from littermates or age-matched wild type and Lfc^−/−^ 8–12-wk-old mice. Mice were bred and housed in accordance with institutional guidelines.

### Generation of immortalized hematopoietic progenitor reporter cell lines

Hematopoietic progenitor cell lines were generated by retroviral delivery of an estrogen-regulated form of HoxB8 as described recently (Redecke et al., 2013; Leithner et al., 2018). Briefly, bone marrow of 6–12-wk-old Lfc*^+/+^* and Lfc^−/−^ mice was isolated and retrovirally transduced with an estrogen-regulated form of the HoxB8 transcription factor. After the expansion of immortalized cells, lentiviral spin infection (1,500 *g*, 1 h) was performed in the presence of 8 µg/ml Polybrene and the lentivirus coding for fluorescent expression construct of interest. Following transduction, cells were selected for stable virus insertion using 10 µg/ml Blasticidin for at least 1 wk. Cells expressing fluorescent reporter constructs were sorted using fluorescence activated cell sorting (FACS; Aria III; BD Biosciences) before migration experiments.

### Dendritic cell culture

The culture was started either from the freshly isolated bone marrow of 6–12-wk-old mice with C57BL/6J background (wild type, Lfc^−/−^, or Lifeact-GFP; Riedl et al., 2010) as described earlier (Lutz et al., 1999) or from stable hematopoietic progenitor cell lines after washing out estrogen. DC differentiation was induced by plating 2 × 10^6^ cells (bone marrow) or 2 × 10^5^ cells (progenitor cells) in complete media (RPMI 1640 supplemented with 10% FCS, 2 mM L-glutamine, 100 U/ml penicillin, 100 µg/ml streptomycin, and 50 µM β-mercaptoethanol; all purchased from Invitrogen) containing 10% granulocyte-monocyte colony-stimulating factor (supernatant from hybridoma culture). To induce maturation, cells were stimulated overnight with 200 ng/ml lipopolysaccharide from *Escherichia coli* 0127:B8 (Sigma) and used for experiments on days 9 and 10.

### In situ migration assay

6–8-wk-old female C57BL/6J mice were sacrificed and individual ear sheets separated into dorsal and ventral halves as described previously (Pflicke and Sixt, 2009). Cartilage-free ventral halves were incubated for 48 h at 37°C, 5% CO_2_ with ventral side facing down in a well plate filled with complete medium. The medium was changed once 24 h after incubation start. If indicated, pharmacological inhibitors were added to the medium. Ear sheets were fixed with 1% PFA followed by immersion in 0.2% Triton X-100 in PBS for 15 min and three washing steps of 10 min with PBS. Unspecific binding was prevented by 60-min incubation in 1% BSA in PBS at room temperature. Incubation with a primary rat-polyclonal antibody against LYVE-1 (Cat. BAF2125; R&D Systems) in combination with rat-polyclonal biotinylated anti–MHC-II antibody (Cat. 553622; BD Biosciences) was done for 2 h at room temperature. After three 10-min washing steps with 1% BSA in PBS, consecutive incubation using Alexa Fluor 488–AffiniPure F(ab')_2_ fragment donkey anti-rat IgG (H+L; Cat. 712–546-150; Jackson ImmunoResearch) secondary antibody and streptavidin-Cy3 secondary antibody (Cat. S6402; Sigma) was done. Samples were incubated in the dark for 45 min with the first secondary antibody, followed by 10-min washing in 1% BSA in PBS, and then with the second secondary antibody. Samples were mounted on a microscope slide with ventral side facing up, protected with a coverslip, and stored at 4°C in the dark.

To determine the distance between the lymphatic vessels and DCs, a mask was created by manually outlining lymphatic vessels depending on Lyve-1 staining and segmenting cells according to their fluorescence intensity. The distance between cells and lymphatic vessels was quantified using a custom-made Matlab script, which determines the closest distance from the segmented cells to the border of the lymphatic vessel binary image. Image borders were excluded from the analysis.

### In vitro collagen gel migration assay

Custom-made migration chambers were assembled by using a plastic dish containing a 17-mm hole in the middle, which was covered by coverslips on each side of the hole. 3D scaffolds consisting of 1.73 mg/ml bovine collagen I were reconstituted in vitro by mixing 3 × 10^5^ cells in suspension with collagen I suspension buffered to physiological pH with Minimum Essential Medium and sodium bicarbonate in a 1:2 ratio. To allow polymerization of collagen fibers, gels were incubated 1 h at 37°C, 5% CO_2_. Directional cell migration was induced by overlaying the polymerized gels with 0.63 µg/ml CCL19 (R&D Systems) diluted in complete media. To prevent drying out of the gels, migration chambers were sealed with Paraplast X-tra (Sigma). The acquisition was performed in 60-s intervals for 5 h at 37°C, 5% CO_2_. A detailed description of the experimental procedure can be found elsewhere (Sixt and Lämmermann, 2011).

### Analysis of y-displacement

Quantification of y-displacement yielded average migration speed of the entire cell population and was performed using a custom-made script for ImageJ as described earlier (Leithner et al., 2016). Briefly, raw data image sequences were background corrected, and particles smaller and bigger than an average cell were excluded. For each time point, the lateral displacement in y-direction was determined as the best overlap with the previous frame and divided by the time interval between frames, yielding the y-directed migration speed of an entire cell population. The respective script can be shared upon request.

### Migration within micro-fabricated polydimethylsiloxane (PDMS)–based devices

Generation of PDMS-based devices and detailed experimental protocols can be found elsewhere (Leithner et al., 2016; Renkawitz et al., 2018). Photomasks were designed using Coreldraw X18, printed on a quartz photomask (1 µm resolution; JD Photo data), followed by a spin coating step using SU-8 2005 (3,000 rpm, 30 s; Microchem) and a prebake of 2 min at 95°C. The wafer was then exposed to ultraviolet light (100 mJ/cm^2^ on an EV Group Germany mask aligner). After a postexposure bake of 3 min at 95°C, the wafer was developed in propylene glycol methyl ether acetate. A 1-h silanization with Trichloro(1H,1H,2H,2H-perfluorooctyl)silane was applied to the wafer. The devices were made with a 1:10 mixture of Sylgard 184 (Dow Corning), and air bubbles were removed with a desiccator. The PDMS was cured overnight at 85°C. Microdevices were attached to ethanol-cleaned coverslips and incubated for 1 h at 85°C after plasma cleaning. Before the introduction of cells, devices were flushed and incubated with complete medium for at least 1 h. Chemokine gradients were visualized by the addition of similar-sized (10 kD; Sigma) FITC-conjugated dextran since their diffusion characteristics are similar (Schwarz et al., 2016). Dimensions of microchannels were 4 x 8 μm (height x width) with the path choice assay and the single-constriciton microchannels containing contrictions of 2 μm, 3 μm, 4 μm, and 5 μm. Pillar arrays had a height of 5 μm.

### In vitro under-agarose migration assay

To obtain humid migration chambers, a 17-mm plastic ring was attached to a glass-bottom dish using Paraplast X-tra (Sigma) to seal the attachment site. For under-agarose migration assay, 4% Ultra Pure Agarose (Invitrogen) in nuclease-free water (Gibco) was mixed with phenol-free RPMI-1640 (Gibco) supplemented with 20% FCS, 1× Hanks’ buffered salt solution, pH 7.3, in a 1:3 ratio. Ascorbic acid was added to a final concentration of 50 µM, and a total volume of 500 µl agarose mix was cast into each migration chamber. After polymerization, a 2-mm hole was punched into the agarose pad, and 2.5 µg/ml CCL19 (R&D Systems) was placed into the hole to generate a soluble chemokine gradient. Outer parts of the dish were filled with water followed by 30-min equilibration at 37°C, 5% CO_2_. The cell suspension was injected under the agarose opposite the chemokine hole to confine migrating DCs between the coverslip and the agarose. Before the acquisition, dishes were incubated at least 2 h at 37°C, 5% CO_2_ to allow recovery and persistent migration of cells. During acquisition, dishes were held under physiological conditions at 37°C and 5% CO_2_.

### Immunofluorescence

For fixation experiments, a round-shaped coverslip was placed in a glass-bottom dish before casting of agarose and injection of cells. Migrating cells were fixed by adding prewarmed 4% PFA diluted in cytoskeleton buffer, pH 6.1 (10 mM MES, 150 mM NaCl, 5 mM EGTA, 5 mM glucose, and 5 mM MgCl_2_) directly on top of the agarose. After fixation, the agarose pad was carefully removed using a coverslip tweezer followed by 20-min incubation in 0.5% Triton X-100 in PBS and three subsequent washing steps of 10 min with TBS containing 0.1% Tween-20 (Sigma). Samples were blocked to prevent unspecific binding by incubating them 60 min in blocking solution (5% BSA, 0.1% Tween-20 in TBS). Immunostainings were performed consecutively by 2-h incubation with rat monoclonal anti–α-tubulin (Cat. MCA77G; AbD Serotec), mouse anti–phospho-MLC 2 (S19; Cat. 3675S; Cell Signaling), mouse anti–γ-tubulin (Cat. Ab11317; Abcam), rabbit anti-acetylated α-tubulin (Cat. T6793; Sigma), or rabbit anti-moesin (Cat. 3150; Cell Signaling), followed by 3 × 10–min washing with blocking solution and 30-min incubation using species-specific Alexa Fluor 488–AffiniPure F(ab')_2_ or Alexa Fluor 647–AffiniPure F(ab')_2_ Fragment IgG (H+L; both Jackson ImmunoResearch) secondary antibodies. After incubation, washing was done at least three times for 5 min each. Samples were conserved in a nonhardening mounting medium with DAPI (Vector Laboratories) and stored at 4°C in the dark.

### Immunodetection of whole-cell lysates

3 × 10^5^ cells were serum starved for 1 h, followed by drug treatment. After harvesting, the cell pellet was snap-frozen and lysed using RIPA buffer (Cell Signaling) to which 1 mM phenylmethanesulfonyl fluoride was added before usage. Samples were supplemented with LDS Sample Buffer and Reducing Agent (both Invitrogen) and incubated for 5 min at 90°C before loading on precast 4%–12% Bis-Tris acrylamide gel (Invitrogen). Subsequently, samples were transferred to nitrocellulose membrane using the iBlot system (Invitrogen) and blocked for 1 h in 5% BSA in TBS containing 0.01% Tween-20. For whole-cell lysate protein detection, the following antibodies were used: rabbit anti–phospho-MLC 2 (S19; 1:500; Cat. 3671; Cell Signaling), rabbit anti–MLC 2 (1:500; Cat. 8505; Cell Signaling), rabbit anti–GEF-H1 (1:500; Cat. 4076; Cell Signaling), rabbit anti–phospho-Ezrin/Radixin/Moesin (ERM)(1:500; Cat. 3141; Cell Signaling), rabbit anti-ERM (1:500; Cat. 3142; Cell Signaling), and mouse anti-GAPDH (1:1,000; Cat. AHP1628T; BioRad). As secondary antibodies, HRP-conjugated anti-rabbit (Cat. 170–6515; BioRad) and anti-mouse (Cat. 170–6516; BioRad) IgG (H+L) antibodies were used in 1:5,000 dilutions, and enzymatic reaction was started by addition of chemoluminescent substrate for HRP (Super Signal West Femto). Chemoluminescence was acquired using a VersaDoc imaging system (BioRad). Western blot signals were quantified manually in Fiji by normalization to input values and subsequent comparison of each treatment to signal intensity of steady-state level (i.e., control sample).

### Flow cytometry

Before staining, 1–2 × 10^6^ cells were incubated for 15 min at 4°C with blocking buffer (1× PBS, 1% BSA, and 2 mM EDTA) containing 5 mg/ml α-CD16/CD32 (2.4G2; Cat. 14–0161-85; eBioscience). For surface staining, cells were incubated for 30 min at 37°C with conjugated monoclonal antibodies; mouse α-CCR7-PE (4B12; Cat. 12–1971-80; BD Biosciences), rat α-mouse I-A/I-E-eFluor450 (M5/114.15.2; Cat. 48–5321-82; BD Biosciences), and hamster α-mouse CD11c-APC (N418; Cat. 17–0114-82; BD Biosciences) diluted at the recommended concentration in blocking buffer. Flow cytometry analysis was performed on a FACS CANTO II flow cytometer (BD Biosciences).

### Pharmacological inhibitors

For perturbation of cytoskeletal and myosin dynamics, we used final concentrations of 300 nM nocodazole and 10 µM Y27632 (both purchased from Sigma). Nocodazole was dissolved in DMSO (Sigma) and Y27632 in PBS (Gibco). Control samples were treated with 1:1,000 DMSO if not indicated differentially.

### Fluorescent reporter constructs

Generation of a C-terminal enhanced GFP (eGFP) fusion construct of Lfc was performed by amplifying Lfc from DC cDNA using a NotI restriction site containing forward (5′-ATA​TGC​GGC​CGC​AAT​CTC​GGA​TCG​AAT​CCC​TCA​CTC​GCG-3′) and reverse (5′-ATA​TGC​GGC​CGC​TTA​GCT​CTC​TGA​AGC​TGT​GGG​CTC​C-3′) primer pairs. After NotI digestion, Lfc was cloned into a pcDNA^TM^3.1 backbone containing eGFP using Express Link T4 DNA-Ligase. The correct sequence and orientation of clones were verified by sequencing (Eurofins). The fluorescent plasmid DNA reporter construct coding for EB3-GFP was a kind gift of V. Small (Institute of Molecular Biotechnology, Vienna, Austria). M. Olson (Beatson Institute, Glasgow, United Kingdom) generously provided MLC constructs (either fused to eGFP or RFP; Croft et al., 2005), and EMTB-3xmCherry constructs were a kind gift of W. M. Bement, University of Wisconsin (Miller and Bement, 2009). Gateway cloning technology was employed to generate lentivirus from plasmid DNA constructs. Briefly, corresponding DNA segments were amplified using primers containing overhangs with *att*B1 and *att*B2 recombination sites on the 3′- and the 5′-end, respectively. To obtain an EMTB fusion construct carrying a single mCherry tag, the PCR product was size separated via gel electrophoresis, and only the fragment of corresponding size (EMTB: 816 bp; mCherry: 705 bp) was further processed. Gel purified PCR fragments were inserted into pcDNA221 entry vectors (Invitrogen) via *att*B and *att*P recombination reaction, generating the entry clone. Expression clones were obtained by carrying out the *att*L and *att*R recombination reaction between the entry clone and pLenti6.3 destination vector (Invitrogen). Lentivirus production was performed by cotransfecting LX-293 cells (Chemicon) with the expression clone of interest in conjunction with pdelta8.9 (packaging plasmid) and pCMV-VSV-G (envelope plasmid; plasmids were a gift from Bob Weinberg, Massachusetts Institute of Technology, Cambridge, MA; Stewart et al., 2003). The supernatant of virus-producing cells was harvested 72 h after transfection, snap-frozen, and stored at −80°C after sterile filtration.

### Transgene delivery

To induce expression of fluorescently labeled proteins, DCs were transfected according to manufacturer guidelines using the nucleofector kit for primary T cells (Amaxa; Lonza Group). Briefly, 5 × 10^6^ cells were resuspended in 100 µl reconstituted nucleofector solution and transferred to an electroporation cuvette, and a total amount of 4 µg plasmid DNA was added. Cells were transfected by using a protocol specifically designed for electroporating immature mouse DCs (program X-001). After transfection, cells were cultured in 60-mm cell culture dishes in complete media and taken for experiments 24 h after transfection. Due to a low transfection efficiency of primary cells, transfected cells were FACS sorted before experiments using FACS Aria III (BD Biosciences).

### Luminometric RhoA activity assay

RhoA activities were determined using G-LISA RhoA Activation Assay Biochem Kit (Cytoskeleton) according to the manufacturer’s instructions. Briefly, 4 × 10^5^ mature BMDCs were lysed in 70 µl RIPA buffer, and protein concentration was determined using the Precision Red Advanced Protein Assay Reagent (Cytoskeleton). Respective samples were treated with 300 nM nocodazole for 15 min before lysis. All samples were adjusted to a final protein concentration of 0.5 mg/ml. Luminescence signals were measured using a microplate photometer at 600 nm. Wells containing lysis buffer only were used as reference blanks in all experiments.

### Microscopy

During all live-cell imaging experiments, cells were imaged in complete media with or without Phenol red and held under physiological conditions at 37°C, 5% CO_2_ in a humidified chamber. Low magnification bright-field or differential interference contrast (DIC) time-lapse acquisition was performed using inverted routine microscopes (Leica) equipped with phase alternating line cameras (Prosilica) controlled by SVS-Visitek software (Seefeld) using 4×/0.1, 10×/0.22, and 20×/0.3 objectives or an inverted Nikon Eclipse wide-field microscope using a C-Apochromat 20×/0.5 PH1 air equipped with a Lumencor light engine (wavelengths [nm]: 390, 475, and 542/575). For high-magnification live-cell acquisition, either an Andor spinning-disk confocal scanhead installed on an inverted Axio Observer microscope (Zeiss), using a C-Apochromat 63×/1.2 W Korr UV-VIS-IR objective, or a total internal reflection (TIRF) setup consisting of an inverted Axio Observer microscope, a TIRF 488/561-nm laser system (Visitron Systems), and an Evolve EMCCD camera (Photometrics) triggered by VisiView software (Visitron) was chosen. Photoactivation experiments were conducted on an inverted spinning-disk microscope (iMic) using a 60×/1.35 Oil objective. TAMRA-stained or EB3-mCherry–expressing DCs were either untreated or treated with 10 µM PST-1 in the dark and recorded using a 561-nm laser line in 2-s intervals. Photoactivation was performed on directionally migrating cells using a 405-nm laser line (pixel dwell time: 10 ms, interval: 40 s). FRAP calibration was performed on separate samples before each experiment. Acquisition of fixed samples (in situ ear crawl in and immunofluorescence samples) was performed using an upright confocal microscope (LSM700; Zeiss) equipped with a Plan-Apochromat 20×/1.0 W DIC (UV) VIS-IR or a Plan-Apochromat 63×/1.4 Oil objective. Fiji and custom-made Matlab scripts were used for image analysis.

### Statistics

In all boxes in box-whisker plots, boxes extend from 25th to 75th percentiles, and whiskers span from minimum to maximum values. Graphs represent pooled data of several cells (*n*) from independent biological experiments (*N*) as mentioned in the figure legends. Individual experiments were validated separately and only pooled if showing the same trend. For the representation of frequencies, bar charts depict mean values from several independent biological experiments (*N*) ± SD. Statistical analysis was conducted using Prism7 (GraphPad). Unpaired Student’s *t* tests were used for Fig. 2, c–e; Fig. 4 b; Fig. 5 e; Fig. 6 a; Fig. S1 i; Fig. S2, b and d; and Fig. S5, a and b; data distribution was assumed to be normal, but this was not formally tested. D’Agostino Pearson omnibus K2 test was used to test for Gaussian or non-Gaussian data distribution, respectively. One-way ANOVA with Tukey’s test was used for Fig. 3 g, Fig. 6 i, Fig. 7 g, and Fig. S5 k; two-way ANOVA with Sidak’s test for Fig. 6 g, Fig. S4 f, and Fig. S5 d; Kruskal-Wallis with Dunn’s test for Fig. 3 h; Fig. 6, f, j, and k; Fig. 7, e, f, and h; and Fig. S5 l; and unpaired two-tailed Mann-Whitney test for Fig. 6 e.

### Online supplemental material

Online supplemental material includes additional data covering cell migration in diverse matrices and characterization of the microtubule cytoskeleton during dendritic cell migration (Fig. S1), data on perturbation of the microtubule cytoskeleton and subcellular Lfc localization (Fig. S2), details of generating an Lfc-deficient mouse strain (Fig. S3), data on aberrant MLC and moesin localization (Fig. S4), and data characterizing the reduced contractile responses of Lfc-deficient dendritic cells (Fig. S5). Videos 1, 2, 3, 4, 5, 6, 7, 8, 9, and 10 contain examples of actively migrating cells during live cell experiments and provide supporting evidence of how microtubules control cellular shape and coherence in amoeboid migrating cells.
